# Inheritable Silencing of Endogenous Genes by Hit-and-Run Targeted Epigenetic Editing

**DOI:** 10.1016/j.cell.2016.09.006

**Published:** 2016-09-22

**Authors:** Angelo Amabile, Alessandro Migliara, Paola Capasso, Mauro Biffi, Davide Cittaro, Luigi Naldini, Angelo Lombardo

**Affiliations:** 1 San Raffaele Telethon Institute for Gene Therapy (SR-Tiget), IRCCS San Raffaele Scientific Institute, Via Olgettina 58, 20132 Milan, Italy; 2 Vita-Salute San Raffaele University, Via Olgettina 58, 20132 Milan, Italy; 3 Center for Translational Genomics and Bioinformatics, IRCCS San Raffaele Scientific Institute, Via Olgettina 58, 20132 Milan, Italy

**Keywords:** epigenetic editing, permanent gene silencing, gene therapy, DNA methylation, B2M-null cells, CRISPR/Cas9, TALE, DNMT3L, TET1, KRAB-ZFP/KAP1

## Abstract

Gene silencing is instrumental to interrogate gene function and holds promise for therapeutic applications. Here, we repurpose the endogenous retroviruses’ silencing machinery of embryonic stem cells to stably silence three highly expressed genes in somatic cells by epigenetics. This was achieved by transiently expressing combinations of engineered transcriptional repressors that bind to and synergize at the target locus to instruct repressive histone marks and de novo DNA methylation, thus ensuring long-term memory of the repressive epigenetic state. Silencing was highly specific, as shown by genome-wide analyses, sharply confined to the targeted locus without spreading to nearby genes, resistant to activation induced by cytokine stimulation, and relieved only by targeted DNA demethylation. We demonstrate the portability of this technology by multiplex gene silencing, adopting different DNA binding platforms and interrogating thousands of genomic loci in different cell types, including primary T lymphocytes. Targeted epigenome editing might have broad application in research and medicine.

## Highlights

• Repurposing the ERV silencing machinery for targeted epigenetic gene silencing
• Hit-and-run delivery of combinations of engineered transcriptional repressors (ETRs)
• Inheritable and stimulation-resistant silencing of endogenous genes by DNA methylation
• Portable to different cell types and DNA binding platforms and amenable to multiplexing

## Graphical Abstract

**Figure:**
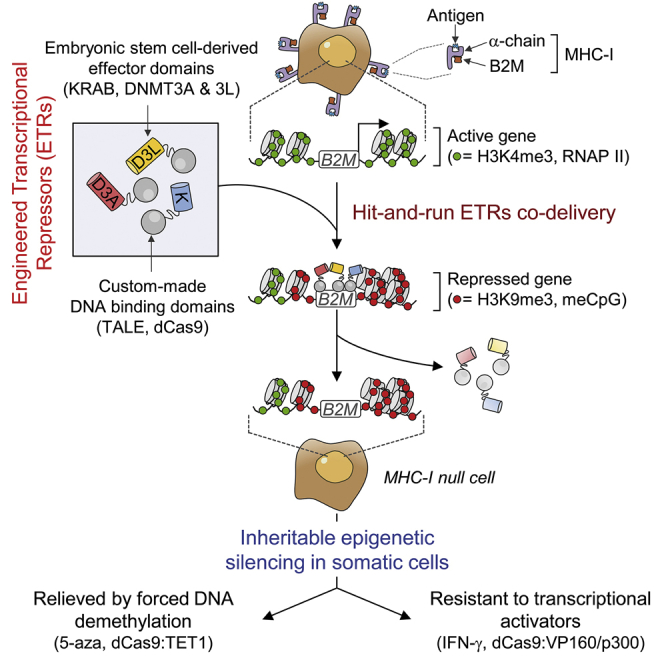

## Teaser

Transient co-expression of engineered transcriptional repressors (ETRs) allows for stable and highly specific epigenetic silencing of endogenous genes, which is amenable to multiplexing and can be reverted by targeted DNA demethylation.

## Introduction

Gene silencing is a powerful strategy to investigate gene function and interrogate the activity of the regulatory genome. It can also be used for therapeutic applications in diseases caused by dominant-negative mutations or conditions in which silencing of a host gene confers resistance to a pathogen (Tebas et al., 2014) or may compensate for an inherited defect in another gene (Bauer et al., 2013). Furthermore, gene silencing can be used to enhance efficacy of cell therapy and for biotechnological applications.

Until now, two main technologies have been used to stably silence gene expression, namely RNAi with short hairpin RNAs (shRNA) (Davidson and McCray, 2011) and gene disruption with artificial nucleases (ANs) (Boettcher and McManus, 2015). The former technology exploits the endogenous microRNA (miRNA) pathway to downregulate expression of a target transcript and mostly requires stable shRNA expression. The latter one exploits the error-prone nature of the non-homologous end joining DNA repair pathway to genetically inactivate the coding frame of the AN-target gene and can be achieved by transient ANs’ expression (Boettcher and McManus, 2015). Although these technologies are widely used in research and are now entering into the clinical arena (Genovese et al., 2014, Tebas et al., 2014, Wittrup and Lieberman, 2015), the partial knockdown by shRNA or the relatively low efficiency of biallelic gene disruption by ANs may limit their efficacy, especially when residual levels of gene activity supports biological function. Furthermore, concerns exist about the specificity and tolerability of each platform, given the potential for off-target activity, which may confound data interpretation and cause toxicity, the possible interference with the endogenous miRNA biogenesis processes by shRNA, and the triggering of apoptosis or differentiation by DNA damage response to AN-induced DNA double-strand breaks (DSBs). Finally, interrogation of non-transcribed regulatory elements, such as promoters or enhancers, is generally not feasible by shRNA or requires extensive tiling by locus-specific arrays of ANs (Canver et al., 2015, Vierstra et al., 2015). Because of these reasons, there is an unmet need for more effective and safer gene-silencing technologies.

A powerful mechanism exploited by eukaryotic cells to permanently repress gene expression is epigenetics, a term encompassing all inheritable changes in the chromatin that affect the function of the genome without altering its primary DNA sequence. Among several mechanisms of epigenetic repression, one of the most characterized is silencing of endogenous retroviruses (ERVs), whose repression is established in the pre-implantation embryo and then maintained throughout development and adult life in most tissues (Friedli and Trono, 2015). Two families of proteins play a pivotal role in this process: the Krüppel-associated box containing zinc-finger proteins (KRAB-ZFPs) and the de novo DNA methyltransferases (DNMTs). KRAB-ZFPs initiate the silencing cascade at ERVs by binding to specific retroviral sequences and recruiting the KRAB associated protein 1 (KAP1). KAP1 in turn complexes with an array of epigenetic silencers, including: SET domain bifurcated 1 (SETDB1) and euchromatic histone-lysine N-methyltransferase 2 (EHMT2, aka G9A), two histone methyltransferases that deposit di- and tri-methylation on lysine-9 of histone H3, and lysine-specific histone demethylase 1 (LSD1, aka KDM1A) and the nucleosome remodeling and deacetylase (NURD) complex, which removes histone H3 lysine-4 methylation and acetyl groups, respectively. These histone-modifying enzymes, together with histone binding and chaperone proteins, including those from the heterochromatin protein 1 (HP1) family, establish a self-reinforcing repressive chromatin state that rapidly spreads from the KRAB-ZFP nucleation site over the ERVs’ regulatory elements, eventually affecting its neighboring genes (Groner et al., 2010). Ultimately, the KAP1-complex recruits the de novo DNMTs 3A or 3B (DNMT3A/3B) (Quenneville et al., 2012), which, in conjunction with the catalytically inactive cofactor DNMT3L, deposit a methyl group on cytosine at CpG dinucleotides, thus permanently locking the repressive state on ERVs. The DNA methylation is then inherited throughout mitosis and somatic cell differentiation without the need for continuous expression of ERV-specific KRAB-ZFPs.

Inspired by this process, we have developed an alternative modality of gene silencing that exploits epigenetics to instruct inheritable repression at selected genomic sites of somatic cells. To this end, we generated engineered transcriptional repressors (ETRs) encompassing a custom-made DNA binding domain (DBD) fused to the effector domain of key players involved in the ERVs’ silencing cascade, including KRAB and the catalytic domain of DNMT3A. Previous studies have shown that ETRs based on these and other epigenetic repressors can be used to silence reporter cassettes or endogenous genes (Keung et al., 2015, Thakore et al., 2016). Silencing, however, required stable expression of the ETR, whereas short-term ETR expression was followed by rapid recovery of the original transcriptional state of the target gene in nearly all treated cells (Hathaway et al., 2012, Kungulovski et al., 2015, Szulc et al., 2006, Vojta et al., 2016). Because of these limitations, we reasoned that combinatorial targeting of multiple effector domains to the regulatory sequences of a given gene of interest might instead mimic the sequential assembly of molecular complexes that are established during early development at ERVs to instruct robust and self-sustaining repressive epigenetic states.

## Results

### Divergent Activity of the KRAB- and the DNMT3A-Based ETRs

**Figure 1:**
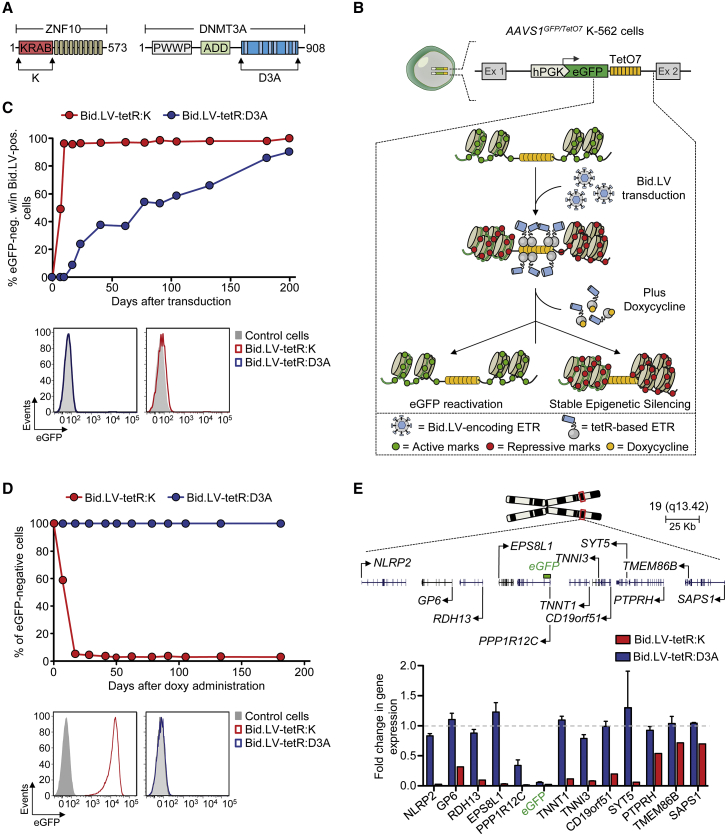
Activity of the KRAB- and DNMT3A-Based ETRs (A) Schematics of the ZNF10 and DNMT3A proteins indicating the KRAB (K) and the catalytic domain of DNMT3A (D3A). (B) Experimental cell model used to assess activity of candidate effector domains. Top drawing shows a K-562 cell clone containing bi-allelic insertion of the hPGK-eGFP.TetO7 cassette into intron 1 of the *PPP1R12C* gene (a.k.a. *AAVS1*). The boxed bottom drawing shows the epigenetic state of the indicated region in the following experimental conditions: (1) in untreated cells, in which the region is decorated by active epigenetic marks and eGFP is expressed; (2) upon transduction with a Bid.LV expressing a tetR-based ETR, whose binding to the TetO7 element leads to deposition of repressive epigenetic marks and silencing of the cassette; (3) and upon conditional release (by doxy administration) of the ETR from the TetO7 element. In this setting, the repressive marks previously deposed by the ETR can be either erased or propagated to the cell progeny by the endogenous cell machinery, thereby leading to transcriptional reactivation or permanent silencing of eGFP expression, respectively. hPGK, human phosphoglycerate kinase gene promoter. (C) Top: graph showing the percentage of eGFP-negative cells within the indicated Bid.LV-transduced cell populations cultured without doxy. Data are represented as mean of *AAVS1*^*GFP/TetO7*^ K-562 cell clones #10 and #27 of Figure S1D. Bottom: representative flow cytometry histograms of the indicated cell populations at termination of the experiment. (D) Top: silenced cells from (C) were sorted and cultured with doxy. The graph shows the percentage of eGFP-negative cells over time. Bottom: histograms of the indicated cell populations at termination of the experiment. (E) Top: schematic of chromosome 19 and zoom on the *AAVS1* locus containing the eGFP-expression cassette. Bottom: gene expression profile of the *AAVS1* locus from eGFP-negative cells transduced with the indicated Bid.LVs. The expression level of each gene was normalized to *B2M* and represented as fold change over a matched, untransduced *AAVS1*^*GFP/TetO7*^ K-562 cell clone (mean ± SEM for Bid.LV-tetR:D3A, n = 3 independent analyses; mean value for Bid.LV-tetR:K, n = 2 independent analyses). See also Figure S1 and Tables S1 and S2.

**Figure S1:**
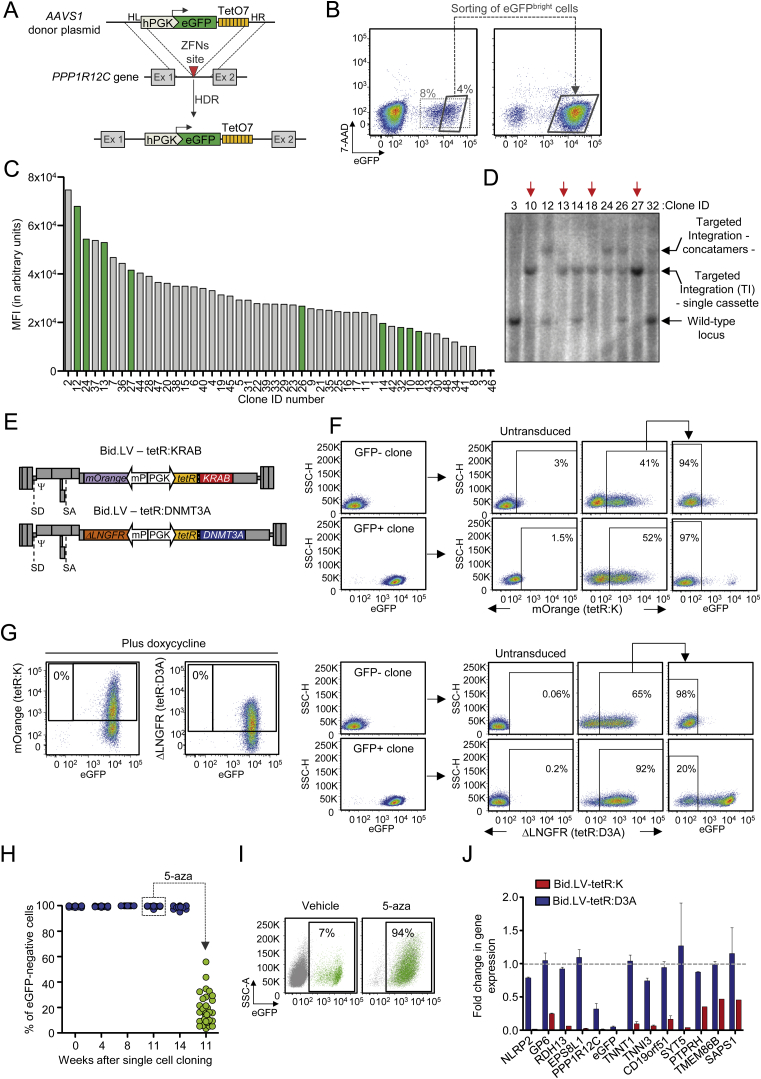
Generation of the *AAVS1*^*GFP/TetO7*^ Reporter Cell Line and Stable Silencing by Targeted DNA Methylation, Related to Figure 1 (A) Schematic of the targeting strategy used to insert the eGFP-expression cassette containing a downstream TetO7 sequence within intron 1 of the *PPP1R12C* gene (aka. *AAVS1*). HL: homology arm left; HR: homology arm right; HDR: Homology Driven Repair; ZFNs: Zinc Finger Nucleases. (B) Gating strategy used to enrich for cells carrying homozygous insertion of the eGFP cassette into *AAVS1*. Left: flow cytometry dot plot showing K-562 cells at day 30 post-transfection with plasmids expressing the *AAVS1*-ZFNs and containing the donor sequence. Right: a representative flow cytometry dot plot of the sorted eGFP^bright^ cells used to derive single-cell clones. (C) Histogram showing the Mean Fluorescence Intensity (MFI) levels of the indicated clonal populations derived from the sorted cells. In green are highlighted the clones selected for further molecular characterization of the integration. (D) Southern blot analysis of the indicated populations performed to identify clones containing homozygous insertion of the eGFP-cassette into *AAVS1*. The red arrows on top of the blot indicate clones selected for subsequent silencing experiments, as they lack signal from the wild-type *AAVS1* allele while they contain Targeted Integration (TI) of the cassette. The expected molecular forms of the *AAVS1* allele – either wild-type or containing the single cassette or its concatamers – are indicated on the right of the blot. (E) Schematics of the Bidirectional Lentiviral Vectors (Bid.LVs) expressing tetR:K and mOrange (top) or tetR:DNMT3A and ΔLNGFR (bottom). Ψ: LV packaging signal; SD: Splicing Donor; SA: Splicing Acceptor; mP: minimal Promoter. (F) Gating strategy used to measure the efficiency of gene silencing within the Bid.LV-transduced cells. An eGFP-negative cell clone, transduced or not with the Bid.LV, was used to set the gate for Bid.LV transduction and eGFP expression. (G) Representative dot plots of *AAVS1*^*GFP/TetO7*^ K-562 cells transduced with the indicated Bid.LVs and cultured in the presence of doxycycline for 200 days. (H) Time-course flow cytometry analysis of 36 independent cell clones derived from the *AAVS1*^*GFP/TetO7*^ K-562 cells transduced with the Bid.LV-tetR:D3A. Cells were grown for 14 weeks with doxycycline. At 11 weeks post-cloning, the populations were treated for 4 days with 5-aza, and then analyzed for eGFP reactivation by flow cytometry. (I) Representative flow cytometry dot plots of cells silenced with the Bid.LV-tetR:D3A and treated or not for 7 days with 5-aza. (J) Gene expression profile of the *AAVS1* locus from eGFP-negative cells transduced with the indicated Bid.LVs. The expression level of each gene was normalized to *HPRT1* and represented as fold change over matched, untransduced *AAVS1*^*GFP/TetO7*^ K-562 cell clone (mean ± SEM for Bid.LV-tetR:D3A, n = 3 independent analyses; mean value for Bid.LV-tetR:K, n = 2 independent analyses).

To quantify the strength and stability of target gene repression imposed by KRAB and the catalytic domain of DNMT3A (Figure 1A; Table S1), we devised an experimental cell model in which release of the ETRs from a reporter expression cassette can be temporally controlled by doxycycline (doxy) administration (Figure 1B). To this end, we first generated a panel of K-562 cell clones with homozygous insertion of an eGFP-expression cassette containing a downstream TetO7 sequence within the ubiquitously transcribed *PPP1R12C* gene (a.k.a. the *AAVS1* locus) (Figures S1A–S1D). We then transduced these *AAVS1*^*GFP/TetO7*^ K-562 cell clones with either of two bidirectional lentiviral vectors (Bid.LVs) (Figure S1E) expressing a marker of transduction together with a fusion protein between the DBD of the tetracycline-controlled repressor (tetR) and KRAB (namely tetR:K) or the catalytic domain of DNMT3A (namely tetR:D3A). Time-course flow cytometry analyses of the transduced cells grown without doxy showed that both ETRs were highly proficient at silencing eGFP expression (Figures 1C and S1F), albeit with different silencing kinetics. On the other hand, when the Bid.LV-transduced cells were maintained in the presence of doxy, neither ETR was able to induce eGFP silencing (Figure S1G), proving the requirement for ETR binding to the cassette for its repression.

We then assessed if the repressive states imposed by the two ETRs were mitotically resistant after release of the repressors from their target cassette and found that the tetR:K-transduced cells rapidly reacquired eGFP expression (Figure 1D). Conversely, the tetR:D3A-tranduced cells remained eGFP-negative for all 180 days of follow-up time (Figure 1D). These results were confirmed by analyzing the progeny of 36 single-cell clones derived from the tetR:D3A-silenced cells (Figure S1H). Of note, exposure of these clones and their parental cell populations to the DNMTs inhibitor 5-aza-2′-deoxycytidine (5-aza) resulted in eGFP reactivation (Figures S1H and S1I), indicating that DNA methylation plays an important role in the maintenance of the repressive state induced by tetR:D3A. We then measured the expression levels of the genes located in a genomic interval of 340 Kb centered on the eGFP-cassette (Figure 1E; Table S2) and found that constitutive binding of tetR:K to its target sequence resulted in substantial downregulation of all genes tested (Figures 1E and S1J). Conversely, only eGFP and, to a lesser extent, the *PPP1R12C* gene—which hosts the reporter cassette in its first intron—were downregulated in cells silenced by tetR:D3A and exposed to doxy (Figures 1E and S1J).

Overall, these data reveal two divergent modes of action of the ETRs. Silencing induced by tetR:K was rapid and robust, spread over the entire analyzed locus, but its effect was fully reversible once the ETR was released from its binding site. On the other hand, silencing induced by tetR:D3A built up with time, was confined around the target site, and was stable over hundreds of cell generations after release of the ETR. The endogenous DNA methylation machinery was required for inheritance of the DNMT3A-induced repressive state.

### Transient Co-delivery of the ETRs Enables Long-Term Silencing

**Figure 2:**
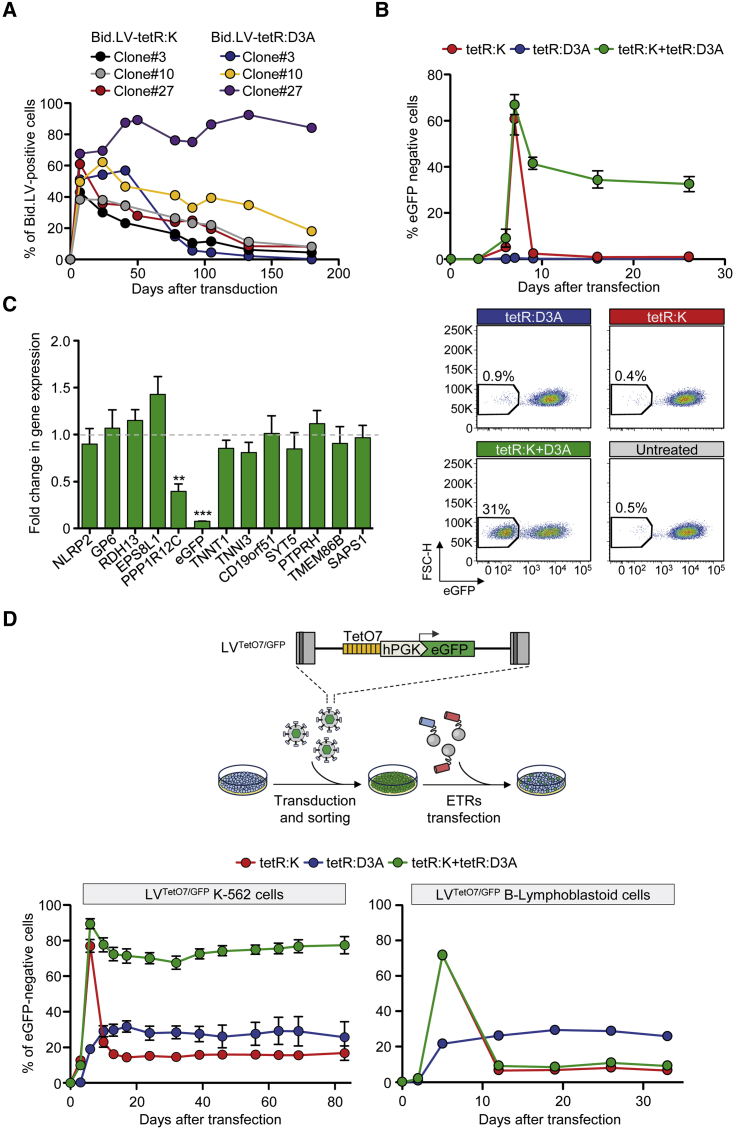
Combination of the KRAB- and DNMT3A-Based ETRs Leads to Synergistic Silencing (A) Time-course analysis of Bid.LV-expressing cells in the indicated *AAVS1*^*GFP/TetO7*^ K-562 cell clones. (B) Top: time-course analysis of *AAVS1*^*GFP/TetO7*^ K-562 cells upon transfection with mRNAs encoding for the indicated ETRs. Data show percentage of eGFP-negative cells (mean ± SEM of Clone #10 and #18 from Figure S1D each transfected in triplicate). Bottom: representative dot plots of the indicated treatments at termination of the experiment. (C) Fold change in the expression levels of the indicated genes in eGFP-negative cells sorted from the double ETRs’ transfected cells. The expression level of each gene was normalized to *B2M* and represented as fold change over untreated K-562 clones. Data are represented as mean ± SEM (n = 3 independent cell sortings from Clone #10 of [B]; statistical analysis by unpaired Student’s t test). (D) Top: schematic of the experimental procedure used to generate the LV^TetO7/GFP^ cell lines and to assess activity of the ETRs. Bottom: time-course analysis of LV^TetO7/GFP^ K-562 cells (left) or B-lymphoblastoid cells (right) upon transfection with mRNA encoding for the ETRs. Data show percentage of eGFP-negative cells (mean ± SEM; n = 3 independent transfections for each treatment condition). See also Figure S2.

**Figure S2:**
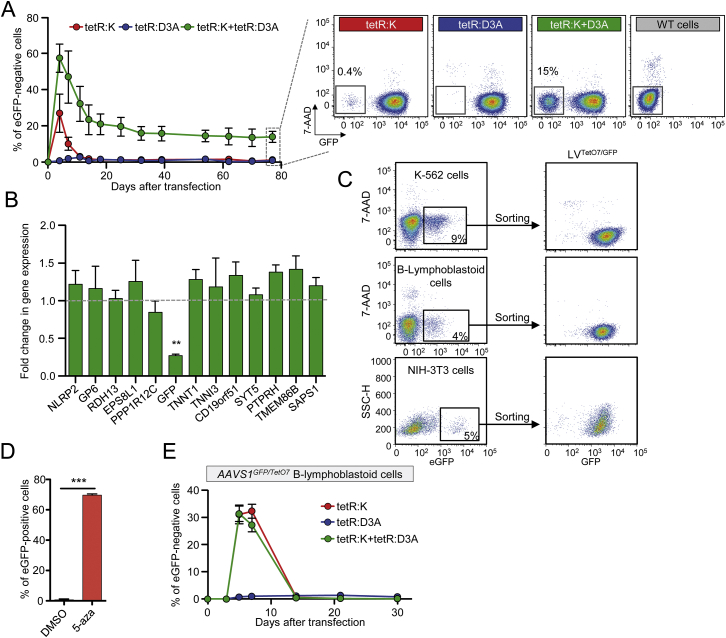
Silencing of the *AAVS1*^*GFP/TetO7*^ Reporter Is Effective in K-562 Cells but Not in B-Lymphoblastoid Cells, Related to Figure 2 (A) Left: time-course flow cytometry analysis of *AAVS1*^*GFP/TetO7*^ K-562 cell Clone #10 upon transfection with plasmids encoding for the indicated ETRs. Data show percentage of eGFP-negative cells (mean ± SEM; n = 3 independent transfections for each treatment condition). Right: representative dot plots of the indicated treatments at termination of the experiment. (B) Fold change in the expression levels of the indicated genes of eGFP-negative cells sorted from the double ETR transfected conditions of (A). The expression level of each gene was normalized to B2M and represented as fold change over matched *AAVS1*^*GFP/TetO7*^ untreated clone. Data are represented as mean ± SEM (n = 3 analyses on sorted cells from 3 independent transfections; statistical analysis by unpaired Student’s t test). (C) Generation of the LV^TetO7/GFP^ cell lines. Representative dot plots showing transduction of K-562 cells (top), B-lymphoblastoid cells (middle) and NIH 3T3 cells (bottom) with LV^TetO7/GFP^ and relative sorting strategies to generate LV^TetO7/GFP^ reporter cell lines (dot plot on the right). (D) Percentage of eGFP-positive cells at day 3 after the indicated treatments (mean ± SEM; n = 3 independent treatments; statistical analysis by unpaired Student’s t test). (E) Time-course flow cytometry analysis of *AAVS1*^*GFP/TetO7*^ B-lymphoblastoid cells upon transfection with in vitro transcribed mRNAs encoding for the indicated ETRs. Data show percentage of eGFP-negative cells (mean ± SEM; n = 3 independent transfections for each treatment condition).

The above results were obtained by stable expression of the ETRs, which may be detrimental to the cells. Indeed, the Bid.LV-positive cells were counter selected in long-term culture in all but one of the previous experiments (Figure 2A). We thus tested transient expression of the individual ETRs and found that neither of them was able to induce long-term silencing of the eGFP-cassette (Figures 2B and S2A), although a short-lasting wave of eGFP repression was seen in up to 60% of the tetR:K-treated cells. On the other hand, transient co-expression of the two ETRs resulted in ∼30% of the cells remaining eGFP silenced long term. Notably, the repressive state induced by the double ETR combination was confined to the eGFP-cassette and its hosting gene (Figures 2C and S2B). These data reveal a synergy between the DNMT3A- and KRAB-based repressors.

We then asked if permanent silencing of the reporter cassette induced by transient ETRs’ co-delivery was a specific feature of the hosting *AAVS1* locus or occurred also when the reporter cassette was randomly distributed throughout the genome. We delivered an eGFP-expression cassette containing the TetO7 sequence semi-randomly into the genome of K-562 cells by standard LV transduction (referred to as LV^TetO7/GFP^ K-562 cells; Figures 2D and S2C) and then transfected the eGFP-positive cells with in vitro transcribed mRNAs encoding for the two ETRs. Time-course flow cytometry analyses showed a rapid and robust spike of eGFP repression in tetR:K treated cells, followed by recovery of eGFP expression in most cells (Figure 2D, bottom left). The tetR:D3A induced a slow and sustained repression in a fraction of the cells. Remarkably, there was a clear synergistic activity of the two ETRs when transiently co-delivered, which resulted in long-term silencing in up to 80% of the treated cells. The long-term silencing induced by the double ETR combination was DNA methylation dependent, as treatment with 5-aza resulted in reactivation of eGFP expression (Figure S2D).

Unexpectedly, however, when we performed similar experiments in human B-lymphoblastoid cells carrying either semi-random LV^TetO7/GFP^ distribution or targeted *AAVS1*^*TetO7/GFP*^ insertion, we failed to observe any long-term effect of the double ETR combination (Figures 2D, bottom right, and S2E). Contrary to the results obtained in K-562 cells, silencing induced by the double ETR combination was transient, with kinetics superimposable to those observed in cells treated with the KRAB-based ETR only. On the other hand, up to 20% of the tetR:D3A-treated cells progressively became eGFP-negative.

Overall, these results indicate that transient co-expression of the two ETRs can instruct stable and confined epigenetic repression of the targeted cassette. This outcome, however, can be constrained in some cell types and by the local chromatin environment.

### Improved Silencing by the Triple Combination of KRAB, DNMT3A, and DNMT3L Effectors

**Figure 3:**
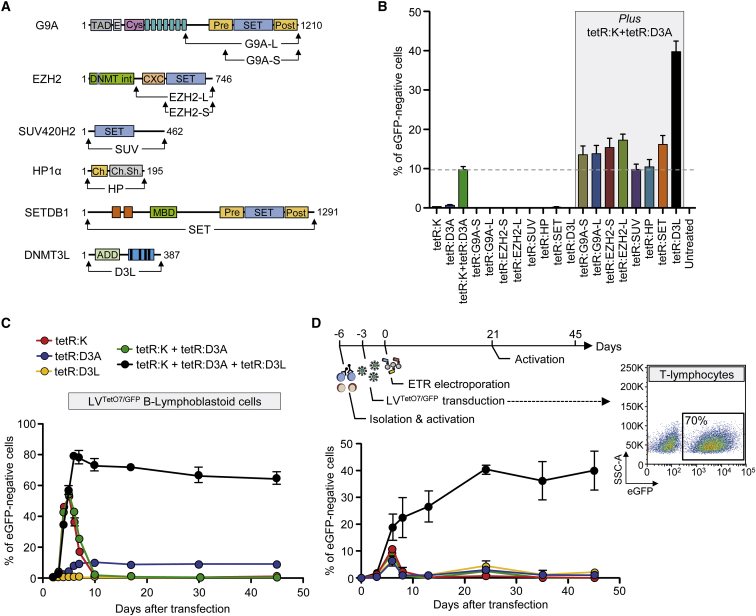
Addition of the DNMT3L-Based ETR to the Double tetR:K tetR:D3A Combination Improves Silencing Efficiency (A) Schematics of the indicated proteins showing the selected effector domains. (B) Histogram showing the percentage of eGFP negative LV^TetO7/GFP^ K-562 cells at 21 days post-transfection with plasmids expressing the indicated ETRs (mean ± SEM; n = 3 of three independent transfections for each treatment condition). (C) Time-course analysis of LV^TetO7/GFP^ B-lymphoblastoid cells upon transfection with mRNAs encoding for the indicated ETRs. Data show percentage of eGFP-negative cells (mean ± SEM; n = 3 independent transfections for each treatment condition). (D) Top: schematic of the experimental procedure used to assess activity of the ETRs in primary T lymphocytes and representative dot plot of LV^TetO7/GFP^ T cells. Bottom: time-course analysis of LV^TetO7/GFP^ T lymphocytes upon transfection with mRNAs encoding for the indicated ETRs. Data show percentage of eGFP-negative cells as calculated by setting to 100% the percentage of eGFP-positive cells in the untransfected LV^TetO7/GFP^ condition (mean ± SEM of two independent blood donors each transfected in duplicate). See also Figure S3 and Table S1.

**Figure S3:**
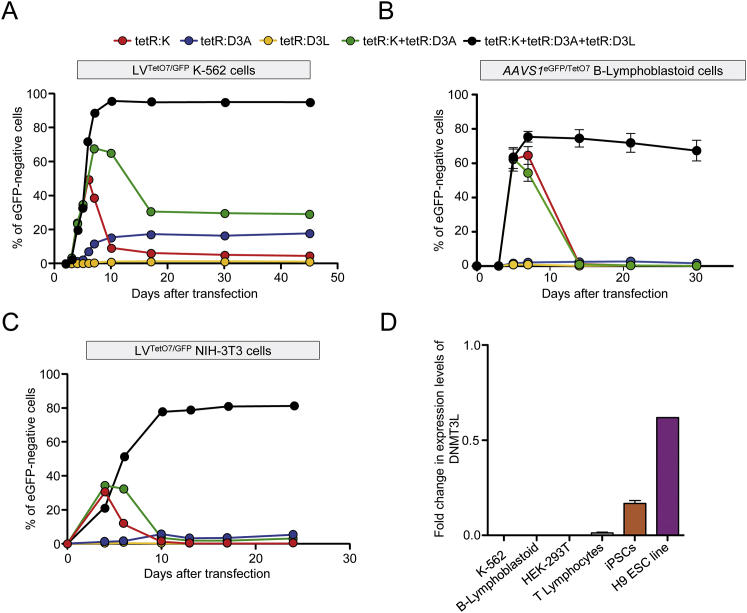
Activity of the Triple ETR Combination in Multiple Cell Types, Related to Figure 3 (A) Time-course flow cytometry analysis of LV^TetO7/GFP^ K-562 cells upon transfection with mRNAs encoding for the indicated ETRs. Data show percentage of eGFP-negative cells (mean ± SEM; n = 3 independent transfections for each treatment condition). (B) Time-course flow cytometry analysis of *AAVS1*^*GFP/TetO7*^ B-lymphoblastoid cells upon transfection with mRNAs encoding for the indicated ETRs. Data show percentage of eGFP-negative cells (mean ± SEM; n = 3 independent transfections for each treatment condition). (C) Time-course flow cytometry analysis of LV^TetO7/GFP^ NIH 3T3 cells upon transfection with mRNAs encoding for the indicated ETRs. Data show percentage of eGFP-negative cells (mean value of 2 independent transfections for each treatment condition). (D) Fold change in the expression level of DNMT3L over HPRT1 in K-562 cells (n = 3), B-lymphoblastoid cells (n = 3), HEK-293T cells (n = 3), human primary T Lymphocytes (n = 3), human induced Pluripotent Stem Cells (iPSC) (n = 7) and H9 human ES cell line (n = 1).

We then asked if adding another effector domain to the KRAB and DNMT3A combination could rescue silencing efficiency in those experimental settings in which the double ETR combination was ineffective. To this end, we selected the following candidates: SETDB1, G9A, HP1α, DNMT3L, enhancer of Zeste homolog 2 (EZH2), and suppressor of variegation 4-20 homolog 2 (SUV420H2) (Figure 3A). We generated tetR-based ETRs containing the effector domains of these human proteins—in some cases of different size (Table S1)—and transiently delivered them either individually or in combination with tetR:K and tetR:D3A in LV^TetO7/GFP^ K-562 cells (Figure 3B). To better detect any increase in the silencing efficiency by the triple ETR combinations, we performed these experiments using non-saturating doses of the ETR-expressing plasmids. When separately expressed, none of the ETRs were able to induce long-term eGFP silencing (Figure 3B). In line with the previous experiments, the double tetR:K and tetR:D3A combination induced long-term eGFP silencing (up to 10%). When the new ETRs were added to this combination, similar or improved silencing efficiencies were measured (Figure 3B). The triple ETR combination containing the DNMT3L-based ETR was the best performing one, showing a 4-fold increase in silencing efficiency over the double tetR:K and tetR:D3A combination. This gain was maintained at higher ETRs doses, reaching up to 98% of long-term eGFP silencing (Figure S3A). Importantly, the triple ETR combination proved to be highly effective also in the B-lymphoblastoid cell lines refractory to the double ETR combination (Figures 3C and S3B). Similarly, the triple, but not the double ETR combination allowed reaching effective silencing in NIH-3T3 mouse cells (up to 80%; Figure S3C) and in human primary T lymphocytes (up to 40%; Figure 3D) containing a mean LV^TetO7/GFP^ copy number of 1 and 6.5 per cell, respectively. Importantly, silencing was resistant to metabolic activation of the T cells after they reached a resting phase upon prolonged culture. Of note, at variance with pluripotent stem cells, none of the cell types used in this study expressed DNMT3L, indicating that the different permissiveness to silencing by the double ETR combination was not due to differential expression of this protein (Figure S3D). Overall, these data show that the triple ETR combination can overcome constrains imposed by cell type or chromatin context, resulting in high efficiencies of silencing across several cell types.

### Stable Epigenetic Silencing of Human Endogenous Genes Using Custom-Made ETRs

**Figure 4:**
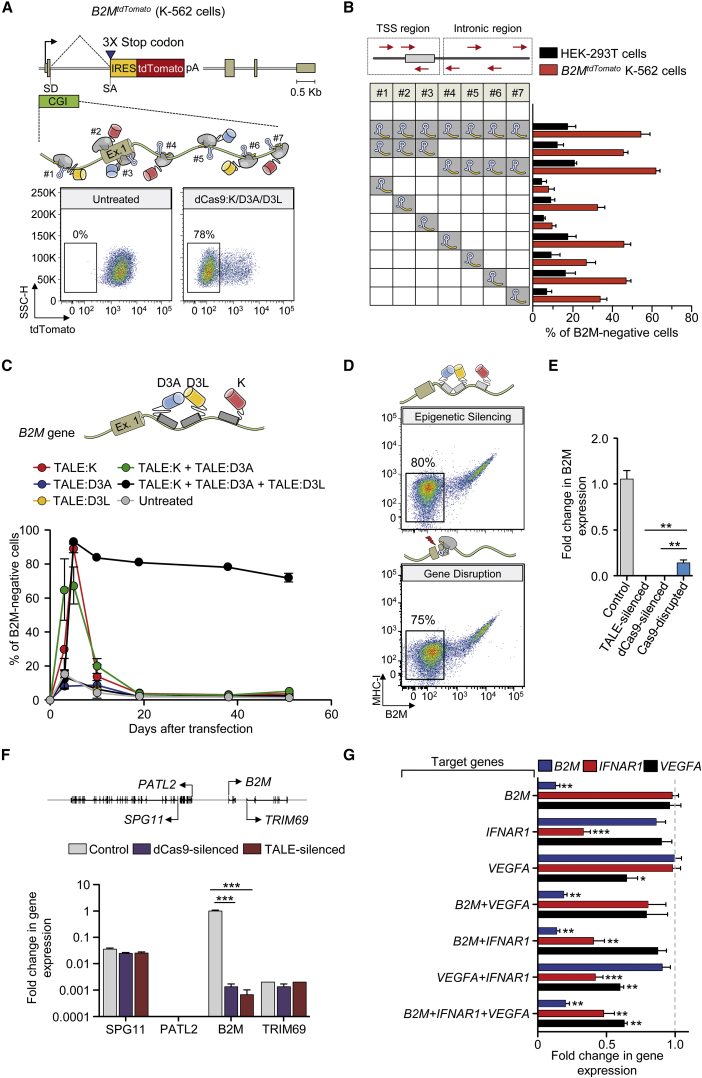
Epigenetic Silencing of Human Endogenous Genes (A) Top: schematics of the *B2M*^*tdTomato*^ gene depicting in the enlarged area the relative order and orientation of binding of dCas9-based ETRs complexed with sgRNAs. CGI, CpG island. Bottom: representative dot plots of *B2M*^*tdTomato*^ K-562 cells either before (left) or after (right) ETR silencing. Analyses at 30 days post-ETR transfection. (B) Silencing activity of the indicated sgRNAs (either in pools or as individual sgRNAs) targeting the promoter/enhancer region of *B2M* (red arrows in the top schematic indicate orientation of the sgRNAs) in *B2M*^*tdTomato*^ K-562 and HEK-293T cells at day 30 post-silencing. Data show percentage of B2M or tdTomato negative cells (mean ± SEM; n = 4 independent transfections for each treatment condition). TSS, transcription start site. (C) Top: schematic of the *B2M* promoter/enhancer region depicting the relative order of binding of the indicated TALE-based ETRs. Bottom: time-course analysis of HEK-293T cells upon transfection with plasmids expressing the indicated ETRs. Data show percentage of B2M-negative cells (mean ± SEM; n = 3 independent transfections for each treatment condition). (D) Representative dot plots of HEK-293T cells at day 30 from the indicated treatments. (E) Fold change in the expression levels of B2M in B2M-negative HEK-293T cells sorted from the indicated conditions. Data are represented as mean ± SEM (n = 3 analyses performed on the indicated populations; for silenced/disrupted cells, each population was sorted from three independent transfection experiments; statistical analysis by unpaired Student’s t test). (F) Top: schematic of the *B2M* locus. Bottom: expression profile of the *B2M* locus of HEK-293T cells sorted from the indicated conditions. The expression level of the indicated genes was normalized to HPRT1 and represented as fold change relative to the B2M levels in untreated cells (calibrator). Data are represented as mean ± SEM (n = 3 analyses performed on the indicated populations; for silenced cells, each population was sorted from three independent transfection experiments; statistical analysis by unpaired Student’s t test). (G) Fold change in the expression levels of the indicated genes in K-562 cells co-transfected or not with plasmids expressing the triple dCas9-based ETRs and sgRNAs targeting the indicated genes. Fold changes are represented relative to the matched untreated control (HPRT1 as normalizer; mean ± SEM; n = 6 independent transfections for each treatment condition). Levels of significance were evaluated with 1-tailed paired t test using values relative to the average of untreated samples. Analyses at 20 days post-transfection. See also Figure S4 and S5 and Table S3.

**Figure S4:**
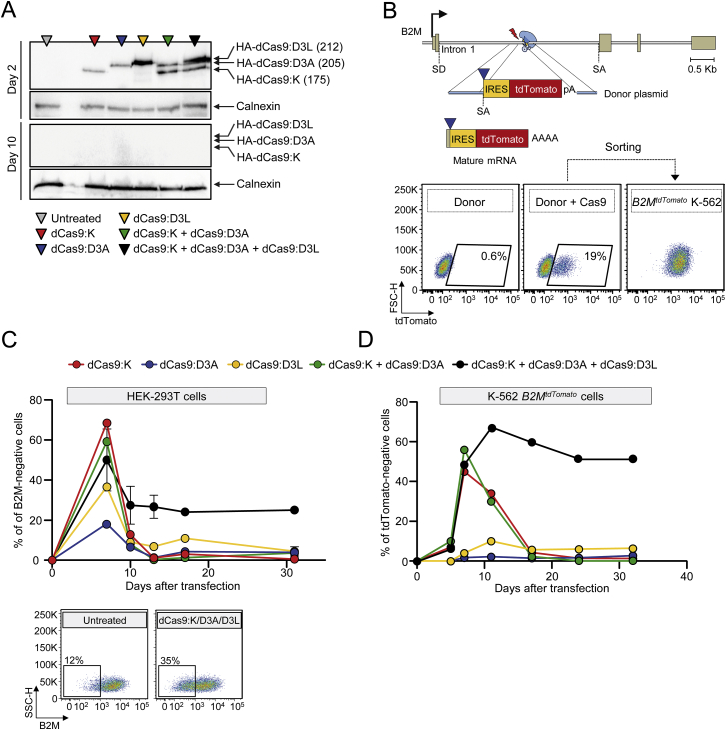
Transient Expression of dCas9-Based ETRs Allows Effective Silencing of the *B2M* Gene, Related to Figure 4 (A) Western blot analysis of K-562 cells 2 (top) or 10 (bottom) days post-transfection with plasmids encoding for the indicated HA-tagged ETRs. Blots were probed with an anti-HA tag or anti-Calnexin antibody, the latter used as loading control. The expected molecular weight (in KDa) of each protein is indicated. (B) Top: schematic of the CRISPR/Cas9-based gene targeting strategy used to insert a tdTomato transgene under the transcriptional control of the *B2M* promoter. Bottom: representative flow cytometry dot plots of K-562 cells transfected as indicated. Analysis at 15 days post-transfection. (C) Top: time-course flow cytometry analysis of HEK-293T cells upon transfection with plasmids encoding for the indicated dCas9-based ETRs and a pool of sgRNAs targeting the *B2M* gene. Data show percentage of B2M-negative cells (mean ± SEM; n = 3 independent transfections for each treatment condition). Bottom: representative dot plots of HEK-293T cells transfected or not with plasmids encoding for the triple dCas9-based ETRs and cognate *B2M*-sgRNAs. Analyses at 30 days post-transfection. (D) Time-course flow cytometry analysis of *B2M*^*tdTomato*^ K-562 cells upon transfection with plasmids encoding for the indicated dCas9-based ETRs and a pool of sgRNAs targeting the *B2M* gene. Data show percentage of tdTomato-negative cells.

To assess if the findings obtained by the triple ETR combination were applicable to an endogenous gene, we initially exploited the CRISPR/Cas9 technology. We separately fused the KRAB, DNMT3A, or DNMT3L domains to the C terminus of a catalytically dead Cas9 (dCas9) and selected seven single guide RNAs (sgRNAs) tiling the promoter/enhancer region of the ubiquitously and robustly expressed β2-Microglobulin (*B2M*) gene. We transiently expressed the dCas9-based ETRs (Figure S4A) together with the *B2M* sgRNAs (Table S3) in K-562 cells engineered to express a tandem dimeric Tomato (tdTomato) transgene from the endogenous *B2M* promoter (referred to as *B2M*^*tdTomato*^ K-562 cells; Figures 4A and S4B) and found that the triple ETR combination induced stable gene silencing in up to 78% of the cells (Figure 4A). Effective gene silencing was also achieved upon deconvolution of the parental sgRNA pool into sub-pools or by using individual sgRNAs (Figure 4B). Similar results, albeit with lower silencing efficiencies (up to 25%), were obtained by silencing *B2M* in HEK-293T cells (Figures 4B and S4C). In both cell types, the individual ETRs and the double dCas9:K and dCas9:D3A combination were ineffective (Figures S4C and S4D). Altogether, these data indicate the feasibility of silencing an endogenous gene with the triple ETR combination.

**Figure S5:**
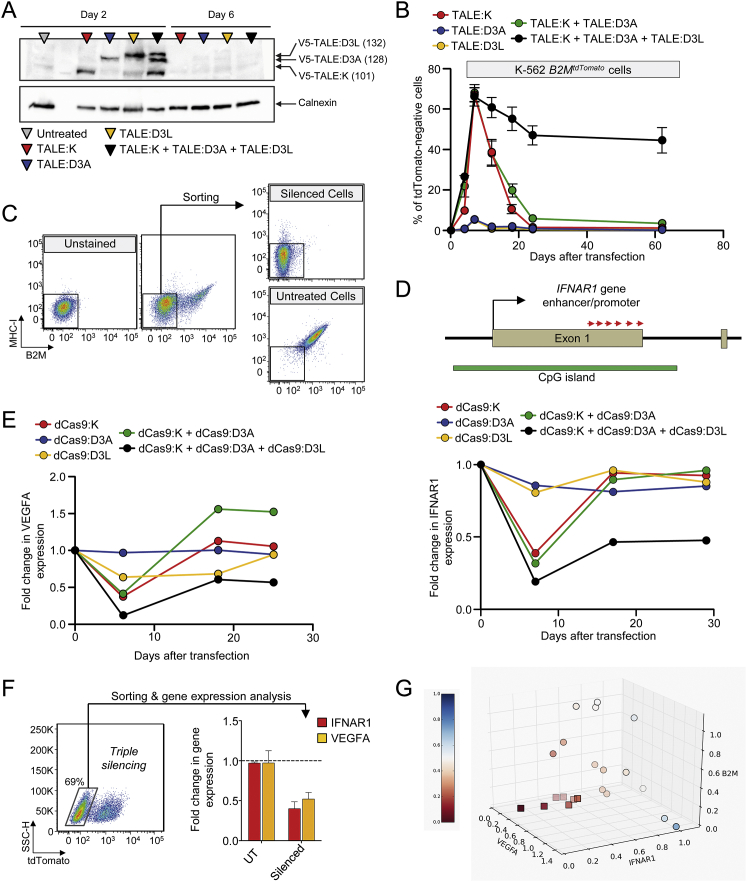
Stable Silencing of Three Human Endogenous Genes by the Triple ETRs Combination, Related to Figure 4 (A) Western blot analysis of K-562 cells 2 (left) or 6 (right) days upon transfection with plasmids encoding for the indicated V5-tagged TALE-based ETRs. Blots were probed with an anti-V5 tag or anti-Calnexin antibody, the latter used as loading control. The expected molecular weight (in KDa) of each protein is indicated. (B) Time-course flow cytometry analysis of *B2M*^*tdTomato*^ K-562 cells upon transfection with plasmids encoding for the indicated TALE-based ETRs. Data show percentage of tdTomato-negative cells (mean ± SEM; n = 3 independent transfections for each treatment condition). (C) Representative dot plots showing the sorting strategy used to obtain B2M-negative HEK-293T cells (top right) from bulk-treated, ETR-silenced cells (middle). Unstained cells are shown (left). (D) Top: schematic of the *IFNAR1* promoter/enhancer region showing the orientation of the sgRNAs (red arrows) and the CGI. Bottom: Graph showing the kinetics of *IFNAR1* silencing (measured as fold change in mRNA levels over untreated cells, *HPRT1* as normalizer) in K-562 cells transfected with plasmids encoding for the indicated dCas9-based ETRs and a pool of 6 sgRNAs targeting the *IFNAR1* promoter/enhancer region. (E) Graph showing the kinetics of *VEGFA* silencing (measured as fold change in mRNA levels over untreated cells, *HPRT1* as normalizer) in K-562 cells transfected with plasmids encoding for the indicated dCas9-based ETRs and a pool of 12 sgRNAs targeting the *VEGFA* promoter/enhancer region. (F) Representative dot plot of *B2M*^*tdTomato*^ K-562 cells treated for multiplex gene silencing (left) and expression analysis of the indicated genes from tdTomato-negative cells (right). The expression level of the indicated genes was normalized to *HPRT1* (mean ± SEM of 2 independent transfections for each treatment condition). Analysis at 25 days post-transfection. (G) Three-dimensional scatterplot depicting the expression levels of *B2M*, *IFNAR1* and *VEGFA* in 21 K-562 cell clones derived from a multiplex gene silencing experiment performed with dCas9-based ETRs and a pool of sgRNAs targeting the three genes. Axes represent the fold change in mRNA levels of the indicated genes over a mean of three untreated cell populations (*HPRT1* as normalizer). Clones with consistent and concurrent downregulation of the three genes are identified by squares. Grouping was performed with Density-Based Spatial Clustering of Applications with Noise (DBSCAN). Points are colored by their euclidean distance from origin of the cartesian space, this corresponding to the ideal perfect triple silencing (0,0,0). Transparency reflects distance from the observer, solid marks are the closest.

Because each dCas9-based ETR exploits the same sgRNAs pool, to avoid DNA binding competition, we uncoupled the three effector domains from the same DBD using the TALE technology. To this end, we targeted the first intron of *B2M* with three different TALE-based ETRs containing the KRAB, DNMT3A, or DNMT3L domain (Figure 4C, top; Table S3). Transient expression of the triple ETR combination in HEK-293T and *B2M*^*tdTomato*^ K-562 cells resulted in up to 73% of long-term gene silencing (Figures 4C, S5A, and S5B). Also in this case, neither the individual ETRs nor the double TALE:K and TALE:D3A combination were able to induce silencing. Of note, the efficiency of silencing with the TALE-based ETR platform was comparable to that obtained by disrupting the *B2M* coding frame through conventional CRISPR/Cas9 technology (Figure 4D). Because of the requirement of B2M for cell-surface exposure of the MHC class I complexes (MHC-I), both the silenced and gene-disrupted cells were negative to staining with pan-MHC-I antibodies (Figures 4D and S5C). The ETR silenced cells expressed ∼500-fold less B2M mRNA than control cells, consistently with the expected transcriptional inactivation of the targeted promoter (Figure 4E). On the other hand, the CRISPR/Cas9 gene-disrupted cells still expressed the B2M mRNA, albeit to 7-fold lower levels than control cells, consistently with nonsense-mediated decay of B2M transcripts bearing a disrupted coding sequence. We then extended the gene expression analysis to a 200 Kb genomic interval centered on the *B2M* gene and found that the only significantly downregulated gene was *B2M*, while expression of its neighboring genes was unaltered (Figure 4F).

In order to assess if the silencing platform was portable to other endogenous genes, we challenged it against the interferon (alpha, beta, and omega) receptor 1 (*IFNAR1*) and the vascular endothelial growth factor A (*VEGFA*) genes, which are highly expressed in K-562 cells. In both cases, silencing was stable and effective (52% or 43% reduction in the mRNA levels of *IFNAR1* and *VEGFA*, respectively) and associated only to treatment with the triple dCas9-based ETR combination (Figures S5D and S5E). Based on these data, we then assessed the feasibility of performing multiplex gene silencing within the same cell using the dCas9-based ETRs. We targeted *B2M*, *IFNAR1*, and *VEGFA* either alone or in combination and found effective and long-term stable co-repression of all genes (Figures 4G and S5F). The repression levels measured in the double or triple gene-silencing conditions were similar to those found by targeting the individual genes and confirmed by analyzing the progeny of 21 single-cell clones derived from the triple-silenced cells (Figure S5G). Overall, these studies show the feasibility of using the triple ETR combination to silence human genes.

### Silencing by the Triple ETR Combination Is Associated to Repressive Epigenetic Marks and Is Resistant to Activation Stimuli

**Figure 5:**
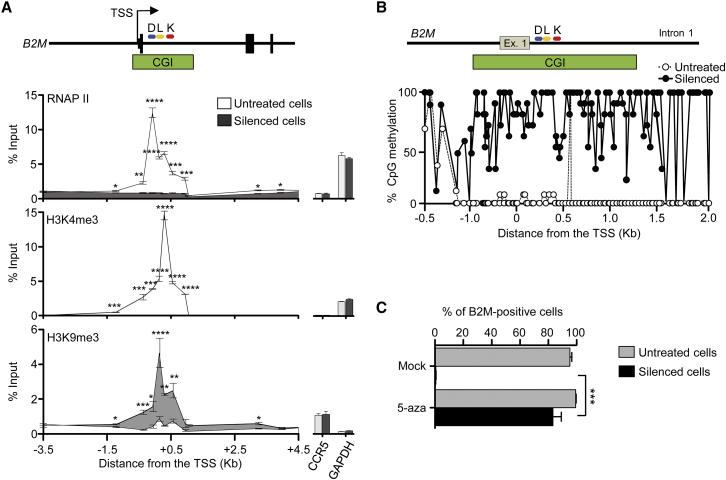
Epigenetic Editing of the ETR Target Gene (A) Chromatin immunoprecipitation followed by qPCR (ChIP-qPCR) analysis for RNAP II, H3K4me3, and H3K9me3 on the *B2M* gene of untreated and silenced HEK-293T cells. Data show fold enrichment over the input (mean ± SEM; n = 3 analyses performed on the indicated populations; for silenced cells, each population was sorted from three independent transfection experiments; statistical analysis by unpaired Student’s t test). Right histograms show percentage of input for an unrelated expressed (*GAPDH*) or not-expressed (*CCR5*) gene. The relative position of the TALE-based ETRs on *B2M* (D, L, and K) is shown. (B) Bisulfite analysis of the *B2M* region depicted in the top schematic from untreated and silenced HEK-293T cells. Data show percentage of CpG methylation. (C) Percentage of B2M positive HEK-293T cells at day 7 after the indicated treatments (mean ± SEM; n = 3 independent treatments; statistical analysis by unpaired Student’s t test).

We compared the epigenetic status of the *B2M* locus between untreated and silenced cells and found that binding of the RNA polymerase II (RNAP II) was reduced to background levels in the silenced cells (Figure 5A). Concomitantly, the promoter/enhancer region of the silenced gene became deprived of the activation mark H3K4me3. This loss was accompanied by acquisition of the repressive mark H3K9me3, whose enrichment was more pronounced at the *B2M* CpG island. At variance with untreated cells, the CpG island of the ETR-silenced cells was highly decorated by de novo DNA methylation (>80% on average) (Figure 5B; Table S4). DNA methylation was also responsible for silencing maintenance, as 5-aza treatment induced re-activation of the *B2M* gene (Figure 5C).

**Figure 6:**
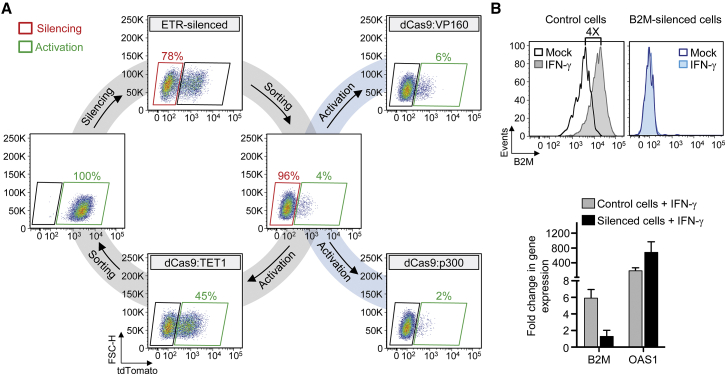
Epigenetic Silencing Is Resistant to External Transcriptional Activation Stimuli and Can Be Reverted by Targeted DNA Demethylation (A) Representative dot plots of *B2M*^*tdTomato*^ K-562 cells treated as indicated (analyses at least at 21 days post-treatment) or upon cell sorting. (B) Top: flow cytometry histograms showing the levels of B2M expression in control or B2M-silenced HEK-293T cells upon exposure or not to IFN-γ. Bottom: expression profile of the indicated genes from the IFN-γ treated cells shown above. The expression level of the indicated genes was normalized to *HPRT1* and represented as fold change relative to untreated cells (calibrator). Data are represented as mean ± SEM (n = 3 independent treatments). See also Figure S6 and Table S4.

**Figure S6:**
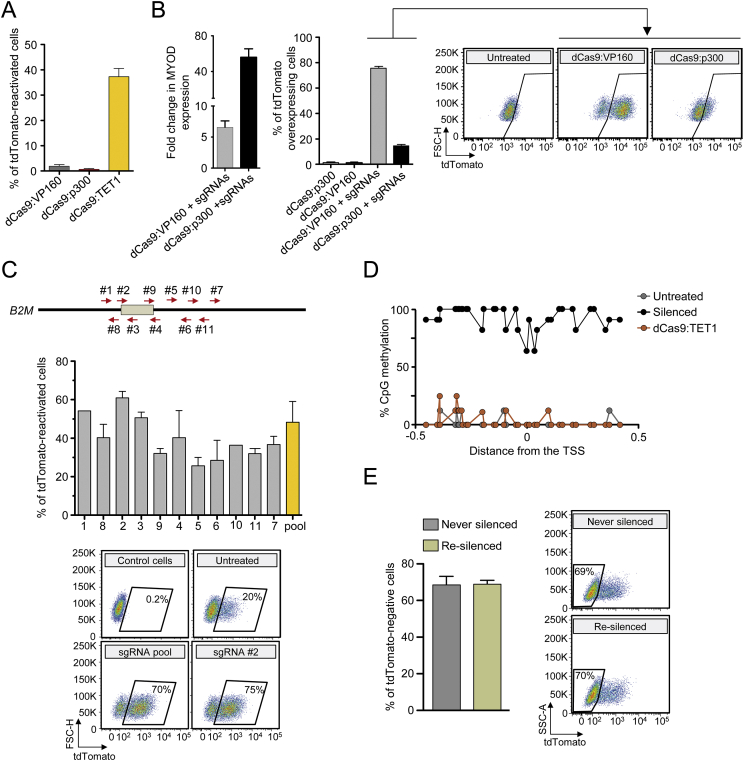
Reactivation of the *B2M* Gene by Targeted DNA Demethylation Is Effective Also with Individual sgRNAs and Is Amenable to Further *B2M* Re-silencing, Related to Figure 6 (A) Histogram showing the percentage of tdTomato-positive cells at 26 days post-transfection with plasmids encoding for a pool of 4 sgRNAs targeting the *B2M* promoter and either dCas9:VP160, dCas9:p300 or dCas9:Tet1 (mean ± SEM; n = 4 independent transfections for each treatment condition). Data are shown upon normalization to the percentage of tdTomato-positive cells present in untreated controls. (B) Left: histogram showing the fold change in mRNA levels of *MYOD* between treated and untretated cells (*B2M* as normalizer). Analysis was performed 5 days post-transfection of HEK-293T cells with plasmids encoding for dCas9:VP160 or dCas9:p300 and a pool of 4 sgRNAs targeting the *MYOD* promoter (mean ± SEM of 2 independent transfections for each treatment condition). Right: histogram showing the percentage of *B2M*^*tdTomato*^ K-562 cells expressing the tdTomato transgene at higher MFI than untreated cells. Analysis was performed 4 days post-transfection with plasmids encoding for dCas9:VP160 or dCas9:p300 with or without a pool of 4 sgRNAs targeting the *B2M* promoter (mean ± SEM; n = 3 independent transfections for each treatment condition). The flow cytometry dot plots show the gating strategy used for the analysis. (C) Top: schematic showing the relative location of the sgRNAs (red arrows) selected to target dCas9:TET1 to the *B2M* enhancer/promoter region. Bottom: histogram showing the percentage of reactivated *B2M*^*tdTomato*^ K-562 cells at 10 days post-transfection with plasmids encoding for the indicated sgRNAs and dCas9:TET1 (mean ± SEM of 2 independent transfections for each treatment condition). Data are shown upon normalization to the percentage of tdTomato-positive cells present in untreated controls. The flow cytometry dot plots show representative samples and the gating strategy used for the analysis. (D) Bisulfite analysis of the *B2M* promoter from untreated (n = 8 PCR sequencings from independent bacterial clones), silenced (n = 11 PCR sequencings from independent bacterial clones) and reactivated (n = 8 PCR sequencings from independent bacterial clones) cells. Data show percentage of methylation of the indicated CpGs. (E) Left: histogram showing the percentage of tdTomato-negative cells 19 days post-transfection with plasmids encoding for the dCas9-based triple ETR combination and for a panel of 4 sgRNAs targeting the *B2M* gene in the two indicated *B2M*^*tdTomato*^ K-562 cell lines (mean ± SEM; n = 3 independent transfections for each treatment condition). Right: flow cytometry dot plots of *B2M*^*tdTomato*^ K-562 cells treated as indicated.

We then exploited the *B2M*^*tdTomato*^ K-562 cell line to quantify the response of the ETR-silenced gene to targeted DNA demethylation or recruitment of engineered transcriptional activators (Figures 6A and S6A). To this end, we exposed the tdTomato-silenced cells to sgRNAs targeting the *B2M* promoter/enhancer region and dCas9 fused to either of the following effectors: (1) the catalytic domain of the DNA demethylase ten-eleven translocation methylcytosine dioxygenase 1 (dCas9:TET1); (2) the transcriptional activator VP160 (dCas9:VP160) (Cheng et al., 2013); and (3) the catalytic core of the acetyltransferase p300 (dCas9:p300) (Hilton et al., 2015). Although both dCas9:VP160 and dCas9:p300 were able to increase expression of a control targeted gene (*MYOD)* or the un-silenced *B2M*^*tdTomato*^ gene (Figure S6B), they induced little—if any—reactivation of the ETR-silenced gene at long-term analysis (Figures 6A and S6A). On the other hand, transient expression of dCas9:TET1 was associated to effective (up to 45%) and long-term stable reactivation of the silenced gene (Figures 6A and S6A), also when using individual sgRNAs (Figure S6C). Reactivation of tdTomato by dCas9:TET1 was accompanied by demethylation of the targeted *B2M* CpG island (Figure S6D). Further exposure of the reactivated cells to the triple ETR combination resulted in re-silencing of *B2M* at efficiencies superimposable to those obtained by treating the parental *B2M*^*tdTomato*^ K-562 cells (Figures 6A and S6E). These data indicate that silencing induced by the triple ETR combination is resistant to artificially recruited transcriptional activators, is stably maintained by DNA methylation, and is amenable to iterative cycles of reactivation and repression by targeted DNA demethylation and methylation, respectively.

Based on these data, we then asked if silencing was also resistant to physiological cell stimulation by exposing control and *B2M*-silenced HEK-293T cells to IFN-γ, a potent inducer of B2M expression. While control cells significantly upregulated B2M expression both at the transcriptional and protein level, no increase in the expression of this gene was measured in the *B2M*-silenced cells (Figure 6B). As expected, IFN-γ caused a significant upregulation of the 2′-5′-oligoadenylate synthetase 1 (*OAS1*) gene (>100-fold), used here as a positive control of IFN-γ exposure, in both cell types (Figure 6B).

### Silencing by the Triple ETR Combination Is Highly Specific

**Figure 7:**
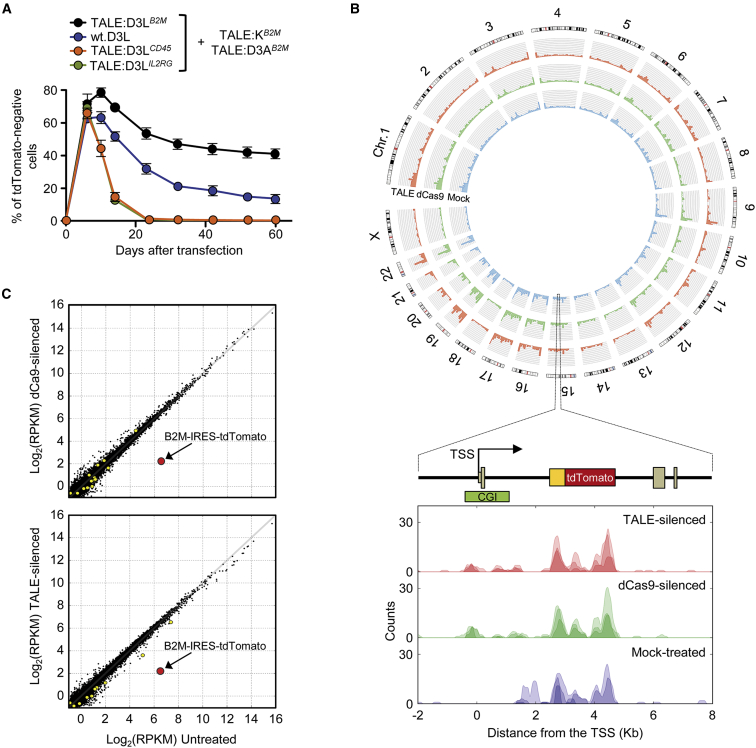
Whole-Genome Profiling of Differential DNA Methylation and mRNA Expression (A) Time-course analysis of *B2M*^*tdTomato*^ K-562 cells upon transfection with plasmids encoding for the indicated TALE-based ETRs with or without WT.D3L. In apex are indicated the TALE target genes. Data show percentage of tdTomato-negative cells (mean ± SEM; n = 3 independent transfections). (B) Top: circos plot showing whole-genome MeDIP-seq profiles of TALE-silenced (red), dCas9-silenced (green), and mock-treated *B2M*^*tdTomato*^ K-562 cells (blue). Bottom: the methylation status of the *B2M*^*tdTomato*^ locus in the indicated samples is shown. Three replicates are represented in each pileup: pileup of aligned reads were smoothed using a Gaussian window. (C) Comparison of expression levels in mock-treated versus dCas9- (top) or TALE-silenced (bottom) cells. Values are expressed in log2 of read per kilobase per million (RPKM) of mapped reads. Black dots represent genes expressed at comparable levels in all conditions; yellow circles represent genes differentially regulated under a FDR < 0.01; red circle represents the B2M-IRES-tdTomato transcript. See also Figure S7 and Tables S5, S6, and S7.

**Figure S7:**
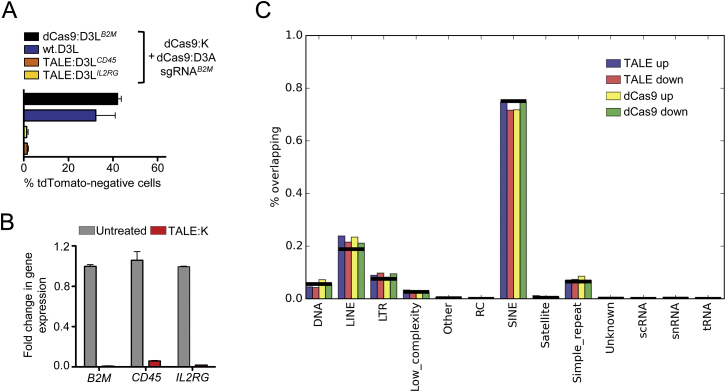
Specificity Analyses of the Triple ETR Combination, Related to Figure 7 (A) Histogram showing the percentage of tdTomato-negative cells at 25 days post-transfection with plasmids encoding for the indicated TALE- and dCas9- based ETRs with or without the wild-type DNMT3L (wt.D3L). Data are shown as mean ± SEM of 3 independent transfections for each treatment condition. (B) Histogram showing the fold change in the expression levels of the indicated genes at day 7 upon transient transfection of plasmids encoding for TALE:K targeting the indicated genes. Data are represented as mean ± SEM of 3 independent transfections for each treatment condition. (C) Enrichment of DMRs (defined at low stringency cutoff: nominal p value < 0.01, logFC > 1) in repetitive elements, defined by RepeatMasker annotation from UCSC Genome Browser track. For each experiment, the ratio of selected DMRs overlapping a specific class of repetitive element was calculated. Horizontal black bars represent the ratio of all analyzable methylated regions (n = 198886) over repetitive elements. None of the ratios was found significantly higher than the expected (test: chi-square).

The DNMT3L-based ETR plays a pivotal role in the silencing platform, being able to consistently increase the repressive activity of the double KRAB- and DNMT3A-based ETR combination by several orders of magnitude. This ETR, however, contains the full-length DNMT3L protein. Beyond its synergistic activity with the other ETRs at their intended target site, this domain may interact with endogenous complexes already engaged in transcriptional repression elsewhere in the genome, potentially enhancing their activity. We thus treated *B2M*^*tdTomato*^ K-562 cells with KRAB and DNMT3A ETRs targeting the *B2M* promoter and DNMT3L either lacking an artificial DBD (wild-type [wt] DNMT3L; wt.D3L) or fused to TALE DBDs targeting the *B2M* or the unrelated *IL2RG* or *CD45* gene promoters (Figures 7A and S7A). As expected, the triple ETR combination targeted to *B2M* led to the highest level of silencing. The wt.D3L also allowed silencing, albeit to a lower efficiency. Strikingly, both of the re-targeted DNMT3L-based ETRs failed to induce *B2M* silencing, indicating that the DBD helps confining the activity of DNMT3L to the targeted locus. Of note, the *IL2RG* and *CD45* targeting TALEs were functional on their intended targets, as shown by effective repression of the two genes once their DBDs were fused to the KRAB domain (Figure S7B).

In order to assess the specificity profile of the triple ETR combination, we performed genome-wide DNA methylation and transcriptional analyses of *B2M*^*tdTomato*^ K-562 cells, either mock-treated or *B2M*-silenced with both TALE- and dCas9-based ETRs. Methylated DNA immunoprecipitation followed by deep-sequencing (MeDIP-seq) analyses showed that the CpG island of the *B2M* gene was the only statistically significant (false discovery rate ≤ 0.01) differentially methylated region (DMR) between mock-treated and both TALE- and dCas9-silenced cells (Figure 7B; Table S5). This region was highly enriched in DNA methylation. In dCas9-silenced cells only, a single Alu sequence mapping to a gene desert region of chromosome 2 also showed increased DNA methylation: further analysis of DMR performed at lower statistical stringency confirmed no enrichment for any class of repetitive element (Figure S7C). Together, these results indicate virtual lack of off-target DNA methylation induced by the silencing platform over the genome. In line with these data, transcriptional profiling by RNA sequencing showed a 24-fold reduction (p value, 6 × 10^−164^; false discovery rate, 8 × 10^−157^) in the expression level of B2M-IRES-tdTomato in cells silenced with either TALE- or dCas9-based ETRs (Figure 7C; Table S6). Few other transcripts were differentially expressed in these analyses, albeit with a much lower fold change and statistical significance than the B2M-IRES-tdTomato transcript: 10 and 14 transcripts were de-regulated in TALE- and dCas9-silenced cells, respectively, four of which were shared between the two datasets (false discovery rate ≤ 0.01). Neither of these transcripts nor the DMR of chromosome 2 of dCas9-silenced cells mapped to a locus containing a computationally predicted off-target site of the TALE DBDs or sgRNA used to target *B2M* (Table S7), indicating that their deregulation might be ascribed to background noise of the analysis or to unknown perturbations caused by the treatment. Overall, these studies support a high degree of specificity of the ETR-silencing platform.

## Discussion

In this study, we repurposed the ERVs’ silencing machinery to develop an efficient and highly specific gene-silencing technology that exploits epigenetics to achieve stable repression of endogenous genes upon transient delivery of combinations of ETRs. We demonstrated the portability of this technology to several genes and different cell types and its versatility by adopting different DNA-binding platforms. Targeted repression could be imposed and relieved from the same promoter via iterative cycles of DNA methylation and demethylation, respectively. The induced repressive epigenetic states were sharply confined to the targeted gene and were resistant to transcriptional activation stimuli.

### Combinatorial ETR Delivery Is Essential for Stable Silencing

Pivotal to these achievements was the adoption of a combinatorial strategy, in which targeted recruitment of the effector domains from KRAB-ZFPs, DNMT3A, and DNMT3L led to the generation of repressive complex(es) capable of instructing self-sustaining epigenetic states. Our studies highlight a previously unreported degree of synergy between the three ETRs. The KRAB/KAP-1 complex may establish a chromatin environment conducive to de novo DNA methylation, which is timely deposited on the targeted gene by the co-delivered DNMT3A-based ETR. Addition of the early developmentally restricted DNMT3L makes silencing highly robust by recreating in somatic cells a powerful repressive complex available to embryonic stem and germinal cells. Mechanistically, DNMT3L could increase the deposition rate, spreading, and stability of DNA methylation that, together with the ensuing chromatin compaction, may contribute to the long-term memory of the silencing phenotypes in the absence of initiating signals. Because silencing could be obtained by using only one sgRNA, we can postulate a stepwise process, in which one ETR instructs a labile repressive state that can then be reinforced by the subsequent timely recruitment of another type of ETR, as well as by amplification through read and write mechanisms exploiting endogenous factors. Once established, DNA methylation may lead to the displacement of activatory transcription factors (TFs) and/or hinder their binding to the gene regulatory elements (Deaton and Bird, 2011). Two lines of evidences support this notion: first, RNAP II was absent throughout the body of the silenced *B2M* gene, indicating failed assembly of the preinitiation complex; and second, targeted recruitment of two different transcriptional activators (VP160 and the catalytic core of p300) or stimulation with IFN-γ were all ineffective at reactivating the silenced gene. Conversely, and in agreement with the primary role of DNA methylation, silencing was reverted either by pharmacological inhibition of the endogenous DNMTs or by targeted recruitment of the TET1 DNA demethylase (Maeder et al., 2013, Wu and Zhang, 2014). The dCas9:TET1 may induce a TF-accessible breach in the methylated regulatory region of the silenced gene and promote its progressive reactivation.

### The Silencing Platform Is Robust and Amenable to Multiplexing

Here, we show robust silencing of three highly expressed genes, namely *B2M*, *IFNAR1*, and *VEGFA*, chosen because of convenient monitoring and the potential therapeutic applications. Notably, a single transient delivery of the triple ETRs’ combination allowed achieving target gene repression in a substantial fraction of treated cells (up to 80%), making further cell enrichment steps dispensable for many applications. When higher purities are needed, however, strategies based on enrichment for highly transfected cells—which are likely those undergoing silencing with higher efficiency—can be used. Our technology allows resetting to background level the expression of genes present at multiple copies per cell, such as the triploid *AAVS1*^*GFP/TetO7*^ locus of K-562 cells and the 6.5 LV^TetO7/GFP^ copies in primary T lymphocytes or multiple genes simultaneously within the same cell. As such, this technology can be readily plugged into the growing armamentarium of targeted transcriptional activators to implement their breadth of applications allowing, for instance, to modulate expression of multiple targets within a given pathway.

Although demonstrated for a limited set of genes, our silencing approach is likely to be broadly applicable to thousands of different loci of both human and mouse cells. This notion comes from experiments performed with semi-randomly integrating LVs, which preferentially insert into actively transcribed genes (Biffi et al., 2013) and whose expression is influenced by the surrounding chromatin environment (Lewinski et al., 2005). These studies shed light on the differential response of the genome to the action of the ETRs and allow assigning the LV-accessible genome to different functional categories: a first one includes loci responsive to KRAB and/or DNMTA3 alone, which likely feature a chromatin environment already poised to the repressive action of each individual ETR, and a second category comprises those loci that are insensitive to any individual ETR but are silenced by DNA methylation when edited by the combination of the two ETRs with or without DNMT3L. Although further studies are needed to clarify which are the key determinants of this differential response, our data suggest that the latter category comprises a larger collection of loci as compared to the former one. Because our studies mostly involved expressed genes, it remains to be elucidated if our findings hold true when trying to imprint stable epigenetic modifications at loci that are repressed in the treated cells but that will become activated at later stages of differentiation and tissue specification. It is tempting to speculate that the pervasive editing capacity of the KRAB-induced repressive complex might lead to effective reprogramming of previously established repressive histone codes deposited by other repressive complexes, such as Polycomb (Schwartz and Pirrotta, 2013), thus facilitating DNA methylation. We expect that the CpG content of a given regulatory sequence might be relevant for the silencing response, as DNA methylation is the main mechanism of stable silencing. To this regard, it is worth mentioning that 70% of annotated mammalian gene promoters are associated with CpG islands (Deaton and Bird, 2011), as in the case of the genes silenced in this study. For CpG-poor regulatory elements, it is possible that methylation of a few key regulatory CpGs may significantly affect their expression (Lal and Bromberg, 2009).

### Targeted Epigenetic Silencing Is Highly Specific

The repressive epigenetic environment induced by the triple ETR platform was sharply confined to the targeted CpG island, as shown by the selective enrichment of both DNA methylation and H3K9me3 at this regulatory element of the *B2M*-silenced gene. The CpG-poor regions flanking the CpG island may act as a boundary to prevent spreading of DNA methylation to nearby regulatory elements, thus sparing proximal genes from repression. It is conceivable, however, that some features of the response to the silencing technology, such as the extent of silencing, its local spreading, and the requirement for all three ETRs, may also vary as we expand the number of investigated loci according to preexisting chromatin environment and genomic architecture.

At the genome-wide level, the silencing technology proved to be highly specific. This positive outcome may reflect the adoption of a combinatorial targeting strategy, which decreases the chances that a given ETR encounters other engineered or endogenous repressors at off-target sites. Furthermore, transient delivery limits the residence time of the ETRs at their DNA binding sites, decreasing also the likelihood of activity at lower affinity off-target sites, which might be particularly relevant for dCas9-based ETRs that share the same sgRNA unless made orthogonal. Notably, this genome-wide analysis, although performed only for the silencing of the *B2M* gene, proves that the ETR effector domains are constrained in their functional activity by the DBD and fail to induce stable repression at other poised genomic sites upon transient delivery. This contention is supported by the finding that ectopic expression of a wild-type DNMT3L complemented at least in part the other two ETRs to induce targeted gene silencing, an activity that was lost when DNMT3L was fused to DBDs targeting unrelated loci. Whereas the specificity profile of ETRs targeting other sites than *B2M* will depend on the specificity of the cognate DBDs/sgRNAs, the lack of off-target activity ascribed to the effector domains once fused to DBDs allows extrapolating our claim on the high specificity of the silencing platform to future applications of this technology.

### Basic and Translational Applications of the Technology

The most notable feature of our silencing strategy is its ability to sharply modify the epigenetic landscape of a given regulatory element, as shown here for the core promoters of three highly transcribed genes. As such, this strategy should provide a versatile tool to dissect the relative contribution of proximal and distal enhancers/promoters in the control of gene expression or to discover novel genetic regulatory elements. To this regard, our strategy might be complementary to powerful in situ saturating mutagenesis assays aiming at identifying key TFs’ binding sites (Canver et al., 2015, Vierstra et al., 2015). While the latter approach requires tiling of the candidate regulatory region with ANs and may suffer from coverage resolution due to limited PAM proximity, our strategy may benefit from using only few ETR docking sites to repress a whole regulatory element. This feature might be of relevance when investigating regulatory elements controlled by multiple TFs, such as super enhancers (Pott and Lieb, 2015). Furthermore, our technology might be useful to investigate the role of *cis-acting* elements encompassing exonic coding regions (Jangi and Sharp, 2014, Mayr, 2015), whose genetic inactivation by ANs induces gene knockout, long non-coding RNAs, whose function is likely insensitive to small nucleotide modifications induced by conventional gene-disruption approaches, and miRNAs, whose random genetic mutagenesis may alter the specificity profile of these broadly acting molecules. Given that an individual sgRNA can drive robust silencing of the ETR target gene, our technology could be used for genome-scale experiments. With respect to previously reported library-based epigenetic silencing strategies (Gilbert et al., 2014), our approach may limit confounding effects due to spreading of the KRAB-induced modifications, an outcome likely to be exacerbated by stable KRAB expression.

The hit-and-run action of the ETRs, which plug into endogenous processes to stably maintain gene silencing without relying on targeted mutagenesis or random genomic insertion, also makes our strategy attractive for the development of biomedical applications. Epigenetic inactivation of regulatory sequences might be readily adopted in gene and cell therapy. A paradigmatic example might be silencing of the erythroid-restricted enhancer of *BCL11A,* which has been proposed as a therapeutic target to reawaken fetal globin expression in patients affected by β-thalassemia or sickle cell disease (Canver et al., 2015, Vierstra et al., 2015). If effective in this context, ETR-mediated silencing would spare hematopoietic stem cells from the risks associated with induction of DSBs by ANs. Concerning the *B2M* gene, one can envision ex vivo engineering of cells of therapeutic relevance, such as lymphocytes, to make them universally transplantable, as proposed for B2M-null cells, but without the need to mutagenize the primary DNA sequence (Riolobos et al., 2013, Wang et al., 2015).

## STAR★Methods

### Key Resources Table

**Table:** 

REAGENT or RESOURCE	SOURCE	IDENTIFIER
**Antibodies**		

Mouse monoclonal PE-conjugated anti-human B2M (clone 2M2)	Biolegend	Cat#316305; RRID: AB_492840
Mouse monoclonal APC-conjugated anti-human MHC-I (clone W6/32)	Biolegend	Cat#311409; RRID: AB_314878
Mouse monoclonal Alexa Fluor 647-conjugated anti-human CD271 (clone C40-1447)	BD Biosciences	Cat#560877; RRID: AB_2033986
Rabbit polyclonal anti-RNA polymerase II CTD repeat YSPTSPS	Abcam	Cat#ab26721; RRID: AB_777726
Rabbit polyclonal anti-histone H3K4me3 antibody	Active Motif	Cat#ab39159; RRID: AB_2615077
Rabbit polyclonal anti-H3K9me3 antibody	Abcam	Cat#ab8898; RRID: AB_306848
Rabbit polyclonal IgG Isotype Control	Abcam	Cat#ab171870
Rabbit polyclonal anti-Histone H3	Abcam	Cat#ab1791; RRID: AB_302613
Mouse monoclonal anti-V5 tag (clone E10)	Abcam	Cat#ab53418; RRID: AB_883403
Mouse monoclonal anti-HA (clone 12CA5)	Roche	Cat#11583816001; RRID: AB_514505
Rabbit Polyclonal Anti-Calnexin	GeneTex	Cat#GTX13504; RRID: AB_368878
Sheep anti-mouse HRP-conjugated IgG	GE Healthcare	Cat# NA9310; RRID: AB_772193
Donkey anti-rabbit HRP-conjugated IgG	GE Healthcare	Cat# NA934; RRID: AB_772206

**Chemicals, Peptides, and Recombinant Proteins**		

Doxycycline	Sigma	A3656; CAS: 2353-33-5
5-Aza-2′-deoxycytidine	Sigma	A3656; CAS:2353-33-5
Recombinant human IFN-gamma protein	R&D Systems	285-IF
cOmplete, Mini, EDTA-free Protease Inhibitor Cocktail	Roche	000000011836170001
Luminata Forte Western HRP substrate	Merck Millipore	WBLUF0100
Dynabeads ClinExVivo CD3/CD28	Invitrogen	402.03D

**Critical Commercial Assays**		

P3 Primary Cell 4D-Nucleofector X Kit S	Lonza	V4XP-3032
SF Cell Line 4D-Nucleofector X Kit S (32 RCT)	Lonza	V4XC-2032
QIAamp DNA Blood Mini Kit	QIAGEN	51104
EpiTect Bisulfite kit	QIAGEN	59104
Rneasy Plus Mini Kit	QIAGEN	74134
TOPO TA Cloning Kit for sequencing	Invitrogen	450071
NextFlex Methyl Sequencing 1 kit	Bioo Scientific	5118-02
IPure kit	Diagenode	C03010011
MagMeDIP kit	Diagenode	C02010021
Ovation Human FFPE RNA-seq Multiplex System 1-8	Nugen	0340
SBS kit v2 200 cycles	Illumina	FC-402-4021

**Deposited Data**		

Raw and analyzed data	This paper	GEO: GSE81826
Human reference genome UCSC build hg19	University of California Santa Cruz	http://hgdownload.soe.ucsc.edu/goldenPath/hg19/bigZips/
Transcript definitions GENCODE v19	GENCODE consortium	http://www.gencodegenes.org/releases/19.html

**Experimental Models: Cell Lines**		

Human: K-562	ATCC	CCL-243
Human: B-lymphoblastoid cells	SR-Tiget	N/A
Human: HEK-293T	SR-Tiget	N/A
Human: Primary T lymphocytes	Blood donors	N/A
Mouse: NHI-3T3	ATCC	CRL-1658
Human: *AAVS1*^GFP/TetO7^ K-562	This paper	N/A
Human: LV^TetO7/GFP^ K-562	This paper	N/A
Human: *AAVS1*^GFP/TetO7^ B-lymphoblastoid cells	This paper	N/A
Human: *AAVS1*^TetO7/GFP^ K-562	This paper	N/A
Human: *B2M*^tdTomato^ K-562	This paper	N/A

**Recombinant DNA**		

pcDNA.Homologies *AAVS1*-hPGK.eGFP.TetO7	This paper	N/A
pBid.LV.mOrange.mhCMV-hPGK.tetR:KRAB	This paper	N/A
pBid.LV.DLNGFR.mhCMV-hPGK.tetR:DNMT3A	This paper	N/A
pLV.TetO7.hPGK.eGFP	Amendola et al., 2013	N/A
pcDNA.CMV.T7.tetR:KRAB	This paper	N/A
pcDNA.CMV.T7.tetR:DNMT3A	This paper	N/A
pcDNA.CMV.T7.tetR:DNMT3L	This paper	N/A
pcDNA.CMV.T7.tetR:G9A-S	This paper	N/A
pcDNA.CMV.T7.tetR:G9A-L	This paper	N/A
pcDNA.CMV.T7.tetR:EZH2-S	This paper	N/A
pcDNA.CMV.T7.tetR:EZH2-L	This paper	N/A
pcDNA.CMV.T7.tetR:SUV	This paper	N/A
pcDNA.CMV.T7.tetR:HP	This paper	N/A
pcDNA.CMV.T7.tetR:SET	This paper	N/A
pcDNA.Homologies *B2M*-SA.3XSTOP.IRES.tdTomato	This paper	N/A
hCas9	Addgene	Plasmid #41815
pcDNA.U6.sgRNA.mod	This paper	N/A
pcDNA.CMV.T7.dCas9:KRAB	This paper	N/A
pcDNA.CMV.T7.dCas9:DNMT3A	This paper	N/A
pcDNA.CMV.T7.dCas9:DNMT3L	This paper	N/A
pAC154-dual-dCas9VP160-sgExpression	Addgene	Plasmid #48240
pcDNA-dCas9-p300 Core	Addgene	Plasmid #61357
pcDNA.CMV.T7.dCas9:TET1	This paper	N/A
Nucleotide sequences of the CRISPRs used in this study; Table S3	This paper	N/A
Golden Gate TALEN and TAL Effector Kit 2.0	Addgene	Kit # 1000000024
TALE-based Epigenetic Repressor Kit	This paper	N/A
pcDNA.CMV.T7.TALE:KRAB^*B2M*^	This paper	N/A
pcDNA.CMV.T7.TALE:DNMT3A^*B2M*^	This paper	N/A
pcDNA.CMV.T7.TALE:DNMT3L^*B2M*^	This paper	N/A
pcDNA.CMV.T7.TALE:KRAB.BGHPA^*CD45*^	This paper	N/A
pcDNA.CMV.T7.TALE:DNMT3L^*CD45*^	This paper	N/A
pcDNA.CMV.T7.TALE:KRAB^*IL2RG*^	This paper	N/A
pcDNA.CMV.T7.TALE:DNMT3L^*IL2RG*^	This paper	N/A
pcDNA.CMV.T7.wt.DNMT3L	This paper	N/A
Nucleotide sequences of the TALEs target sites and the corresponding repeat variable diresidues (RDVs); Table S3	This paper	N/A

**Sequence-Based Reagents**		

Gene expression assays; Table S2	This paper	N/A
Primers used in this study; Table S4	This paper	N/A

**Software and Algorithms**		

Flowjo	FLowJo LLC	http://www.flowjo.com/RRID:SCR_008520
FSC Express 4	De Novo software	https://www.denovosoftware.com/
CRISPR design suite	Hsu et al., 2013	http://crispr.mit.edu/
QUantification Tool for Methylation Analysis (QUMA)	Kumaki et al., 2008	http://quma.cdb.riken.jp/
TALENT 2.0	Doyle et al., 2012	https://tale-nt.cac.cornell.edu/node/add/single-tale
STAR	Dobin et al., 2013	https://github.com/alexdobin/STAR
Rsubread	Liao et al., 2013	https://bioconductor.org/packages/release/bioc/html/Rsubread.html
edgeR	Robinson et al., 2010	https://bioconductor.org/packages/release/bioc/html/edgeR.html
bwa	Li and Durbin, 2010	https://github.com/lh3/bwa
MACS2	Zhang et al., 2008	https://github.com/taoliu/MACS/tree/master/MACS2
bedtools	Quinlan, 2014	http://bedtools.readthedocs.io/en/latest/
cqn	Hansen et al., 2012	http://bioconductor.org/packages/release/bioc/html/cqn.html

### Contact for Reagent and Resource Sharing

Further information and requests for reagents may be directed to, and will be fulfilled by the corresponding author Angelo Lombardo (lombardo.angelo@hsr.it).

### Experimental Model and Subject Details

#### Cell Culture Conditions and Engineering

Human B-lymphoblastoid cells were maintained in RPMI-1640 (Sigma); HEK-293T and K-562 in IMDM (Sigma); NIH/3T3 in DMEM (Sigma). All media were supplemented with 10% Fetal Bovine Serum (FBS) (EuroClone), L-glutamine (EuroClone) and 1% Penicillin/Streptomycin (100 U/ml final concentration; EuroClone). Cells were cultured at 37°C in a 5% CO_2_ humidified incubator. The reporter cell lines with semi-random integration of the TetO7/eGFP cassette were generated by transducing the cells with the LV^TetO7/GFP^ at Multiplicity of Infection (MOI) of 0.1, and then by sorting the eGFP-expressing cells using the MoFlo XDP Cell Sorter (Beckman Coulter). Human primary T lymphocytes were isolated from peripheral blood mononuclear cells of healthy donors by leukapheresis and Ficoll-Hypaque gradient separation. The cells were activated and enriched to purity using magnetic beads conjugated to antibodies against CD3 and CD28 (ClinExVivo CD3/CD28; Invitrogen), following the manufacturer instructions, and grown at a concentration of 1 × 10^6^ cells/ml in RPMI (Sigma) supplemented with penicillin, streptomycin, 10% FBS and 5ng/ml of IL-7 and IL-15 (PeproTech) as previously described (Lombardo et al., 2011). After three days of culture, the cells were transduced with the LV^TetO7/GFP^ at MOI of 10 and then used for subsequent experiments. The use of human primary T cells was approved by the San Raffaele Hospital Bioethical Committee (TIGET PERIBLOOD). The *AAVS1*^*GFP/TetO7*^ and the *AAVS1*^*TetO7/GFP*^ K-562 cells were generated as follows: K-562 cells were co-transfected with (i) a targeting construct containing the hPGK.eGFP-expression cassette (Lombardo et al., 2011) - with the TetO7 either upstream or downstream of it - within homology arms to the *AAVS1* locus, and (ii) in vitro transcribed (IVT) mRNAs encoding for the previously described *AAVS1*-ZFNs (Genovese et al., 2014). A similar targeting strategy was used to generate the *AAVS1*^*GFP/TetO7*^ B-lymphoblastoid cells. Single-cell clones derived from the bulk-targeted eGFP-positive cells were then obtained by limiting dilution plating, and analyzed by Southern blot to confirm targeted integration of the cassette, as previously described (Lombardo et al., 2011). The *B2M*^*tdTomato*^ K-562 cells were generated as follows: K-562 cells were co-transfected with (i) a targeting construct containing the splice acceptor-3xSTOP-IRES-tdTomato-pA cassette within homology arms to intron 1 of *B2M*, (ii) a construct encoding for the catalytically active Cas9, and (iii) a construct expressing the intron 1 *B2M* sgRNA (*B2M*-CRISPR sequence: 5′-AGGCTACTAGCCCCATCAAGAGG-3′). Bulk-targeted, tdTomato-positive cells were then sorted and used as indicated.

### Method Details

#### Lentiviral Vectors and Constructs

Bid.LVs and LV^TetO7/GFP^ were generated from third generation self-inactivating LV transfer constructs (Amendola et al., 2013, Cantore et al., 2015). Cloning information are available upon request. VSG-G pseudotyped LV stocks were prepared as previously described (Cantore et al., 2015). The tetR-based ETRs were generated by replacing the KRAB domain from the tetR:KRAB construct (Groner et al., 2010) with the indicated repressor domains. The amino acid sequences of the effector domains are available in Table S1. The dCas9-based ETRs were generated by replacing the VP160 trans-activator from the plasmid pAC154-dual-dCas9VP160-sgExpression (Addgene No. 48240) (Cheng et al., 2013) with the indicated repressor domains or with the catalytic domain of TET1 (the amino acid sequence of TET1 is available in Table S1). The catalytically active Cas9 (Mali et al., 2013) and the dCas9:p300 (Hilton et al., 2015) plasmids were from Addgene (No. 41815 and No. 61357, respectively). CRISPRs were selected using the online software CRISPRtool (http://crispr.mit.edu) and expressed from the human U6 promoter as fusion transcripts with the previously described tracrRNA^(F+E)^ (Chen et al., 2013). Sequences of the CRISPRs are available in Table S3. TALE-based ETRs were generated using a modified version of the Golden Gate TALEN Kit 2.0a (Addgene Kit#1000000024) (Cermak et al., 2011) containing the following architectural changes: the Golden Gate TALE C- and N-terminal subregions were replaced respectively with the +163 and +63 terminal deletions. These constructs were further adapted to accommodate in frame the repressor domains. We designated this platform as TALE-based Epigenetic Repressor Kit. TALE target sites were selected using the online software TAL Effector Nucleotide Targeter 2.0 (Doyle et al., 2012). The TALE target sites and the corresponding RDVs sequences are available in Table S3.

#### Gene Delivery Procedures and Cell Treatments

IVT mRNAs were produced as previously described (Genovese et al., 2014). Silencing experiments were performed by transducing the indicated cell types with the ETR-expressing Bid.LVs at MOI of 10 or by transfecting them with plasmids or IVT mRNAs encoding for the ETRs (0.5-2 μg of nucleic acid for each tetR- or TALE-based ETR; 1-2 μg of plasmid for each dCas9-based ETR and 125-250ng of plasmid for each sgRNA-expressing plasmid). Reactivation experiments were performed by transfecting the *B2M*^*tdTomato*^ K-562 cells with plasmids encoding for the dCas9-based activators (1.5 to 6 μg for each activator; 250ng of plasmid for each sgRNA-expressing plasmid). For silencing experiments in primary T-lymphocytes, three days after transduction with the LV^TetO7/GFP^, the cells were transfected with 2 μg of IVT mRNA encoding for each of the tetR-based ETRs. Twenty-one days post-transfection, the resting T lymphocytes were activated by polyclonal TCR stimulation via co-culturing the cells with a pool of 6000 rad irradiated PMBCs from unrelated donors and 10000 rad irradiated B-lymphoblastoid cells in the presence of 30ng/mL of anti-CD3 antibody (OKT3; Orthoclone) and 50U/mL of human recombinant IL-2 (PrepoTech). Transfections were performed using the 4D-Nucleofector System (Lonza) and following manufacturer’s instructions for K-562, HEK-293T, NIH/3T3 and T-lymphocytes, or using the pulse program EW-113 and SF solution for B-lymphoblastoid cells. When indicated, the cells were treated with 1 μM of 5-Aza-2-deoxycytidine (AZA, Sigma), 12 μg/ml of doxycycline (Sigma), or 500U/ml of human recombinant IFN-γ (R&D Systems). The AZA- and the IFN-γ−containing media were replaced daily, and the cells were analyzed by flow cytometry respectively at day 4 and 7, or at day 2 and 4.

#### Flow Cytometry and Gene Expression Analyses

Flow cytometry was performed using FACSCanto II or LSR Fortessa (BD Biosciences) and raw data were analyzed using FlowJo (FLowJo LLC) or FSC Express 4 (De Novo software). Immunophenotypic analyses were performed using the antibodies listed in the Key Resource Table. 1–5 × 10^5^ viable cells, as gauged by 7-Aminoactinomicin D (Sigma) exclusion, were analyzed *per* sample. Single- and fluorescence minus one-stained cells were used as controls. Gene expression analyses were performed using the TaqMan Gene Expression assays (Applied Biosystems) listed in Table S2 or with the PCR primers listed in Table S4. For sample preparation, total RNA was extracted from 2-6x10^6^ cells using the RNeasy Mini kit (QIAGEN) and reverse-transcribed using random hexamers according to the SuperScript III First-Strand Synthesis System (Invitrogen) manufacturer’s instructions. Real-time PCR was performed in triplicate (15-100ng of cDNA equivalents *per* sample) using the ViiA 7 Real-Time PCR System (Applied Biosystems) and analyzed using the included software to extract raw data (C_t_) as previously described (Lombardo et al., 2011). Genes with a C_t_ value ≥ 37 were excluded from the analyses. To determine gene expression, we calculated the difference (ΔC_t_) between each gene and a reference gene (*HPRT1*, *B2M* or *GAPDH*). Gene expression results are indicated as fold change to a reference sample, calculated using the 2^–ΔΔCt^ method.

#### Molecular Analyses

Genomic DNA was extracted using the DNeasy Blood & Tissue Kit or QIAamp DNA Mini Kit (QIAGEN). Southern blot and vector copy numbers were performed as previously described (Lombardo et al., 2011). Bisulfite DNA conversion was performed using the EpiTect Bisulfite kit (QIAGEN) according to manufacturer’s instruction. Converted DNA was PCR-amplified using the *B2M* primer pairs listed in Table S4. PCR fragments were agarose gel purified, cloned into the pCR4-TOPO plasmid (Invitrogen) and sequenced using the M13 universal primer. Conversion analyses were performed using the online software QUMA (Kumaki et al., 2008). Chromatin Immunoprecipitation followed by qPCRs (ChIP-qPCRs) were performed as previously described (Lombardo et al., 2011) using 5-10μg of the anti-RNAP II CTD repeat YSPTSPS antibody (Abcam ab26721), anti-histone H3K4me3 antibody (Active Motif 39159) and anti-H3K9me3 antibody (Abcam ab8898) *per 25-*50μg of chromatin. Anti-Histone 3 antibody (Abcam ab1791) was used as normalizer for histone modifications and matching IgG isotype was used as unrelated IgG control (Abcam). Sequences of the primers used for the ChIP-qPCR analyses are listed in Table S4. The percentage of input for each investigated site was calculated by the ΔC_t_ method using the Input as normalizer. A genomic site in *GAPDH* and another in *CCR5* were used as positive and negative controls for the analyses, respectively.

#### RNA Sequencing Analysis

For the RNA sequencing experiments, *B2M*^*tdTomato*^ K-562 cells were transfected in triplicate with plasmids encoding for: (i) the TALE-based ETRs for the *B2M* gene; (ii) the dCas9-based ETRs *plus* the sgRNA targeting the *B2M* sequence 5′-GCGTGAGTCTCTCCTACCCT-3′; (iii) the dCas9 protein without any effector domain *plus* the sgRNA targeting the *B2M* sequence 5′-GCGTGAGTCTCTCCTACCCT-3′ (the latter two samples were used as mock-treated controls). Silenced cells were sorted at day 25 post-transfection (purity ≥ 90%) and used for subsequent analyses. RNA was extracted using the RNeasy Mini kit (QIAGEN) according to manufacturer’s instruction. Amplification of cDNA from total RNA (starting amount 600-1000 ng/ul *per* sample) was performed using the Ovation Human FFPE RNA-seq Library System (Nugen), and cDNA was fragmented with E220 COVARIS ultrasonicator (Covaris). Library quantification and quality control was performed on Bioanalyzer2100 (Agilent). Barcoded libraries were pooled, denatured, and diluted to a 7 pM final concentration. Cluster formation was performed on board of HiSeq 2500 Rapid Mode flow cell (Illumina). Sequencing By Synthesis (SBS) was performed according to HiSeq PE protocol v2 (Illumina) on HiSeq 2500 system (Illumina) set to 200 cycles, yielding an average of 30M reads/sample. Read tags were aligned to reference genome hg19 using STAR v 2.3.0 (Dobin et al., 2013), with default parameters. Features were counted using Rsubread package (Liao et al., 2013), with gencode v19 (Harrow et al., 2012) as gene model. Alignment on tdTomato sequence and its quantification were performed separately. Feature counts, summarized at gene level, were normalized with TMM (Robinson and Oshlack, 2010). A filter of at least one count per million (cpm) in at least 3 samples was used to discard low-expressed genes. Differential gene expression was evaluated with Negative Binomial Generalized log-linear model implemented in edgeR (function glmFit) (Robinson et al., 2010). A threshold of 0.01 was set on adjusted p-values (Benjamini-Hochberg correction, BH) to retain differentially regulated genes. RPKM values used in Figure 7C were also calculated using edgeR.

#### Methylated DNA Immunoprecipitation Followed by Deep-Sequencing Analysis

For each sample used in the RNA sequencing experiments, 500 ng of genomic DNA extracted with QIAamp DNA Mini Kit (QIAGEN) was sonicated with E220 COVARIS ultrasonicator (Covaris) and sequencing libraries were prepared using NextFlex Methylseq kit 1 (Bioo Scientific). After adaptor ligation step, samples were pooled and immunoprecipitated using a monoclonal antibody directed against 5-methylcytosine (MagMeDIP kit, Diagenode). Enriched and control libraries (not immumoprecipitated) were purified using the IPure kit (Diagenode). Enrichment efficiency was evaluated by quantitative real-time PCR using provided internal controls (spiked-in DNA from *A. thaliana*). Libraries were amplified following NextFlex Methylseq kit 1 protocol. After library quantification and quality control on Bioanalyzer2100, SBS was performed according to HiSeq PE protocol v2 (Illumina) on HiSeq 2500 system (Illumina) set to 200 cycles, yielding an average of 30M reads/sample. Read tags were aligned to the reference genome hg19 using bwa v 0.7.5 (Li and Durbin, 2010). Peaks were identified using MACS v 2.0.10 (Zhang et al., 2008) allowing for broad peaks identification (–slocal = 0,–llocal = 500000). Peaks identified in different samples were unified using bedtools multiintersection tool (Quinlan, 2014) with clustering option. Read counts over the final peak list were calculated using bedtools, discarding duplicated reads. In order to evaluate differential methylation, we adopted Generalized log-linear model implemented in edgeR (function glmFit), normalization was performed using Conditional Quantile Normalization (Hansen et al., 2012) in order to model region-wise GC-content. A threshold of 0.01 was set on BH adjusted p-values to retain differentially methylated regions. Analysis of repeated sequences was performed as follows: MeDIP results were filtered for nominal p value < 0.01 and absolute logFC > 1 so that four datasets were produced (dCas9 up/down and TALE up/down). We counted the number of intersections of the regions in these datasets with all classes of repeats annotated in hg19 genome in the RepeatMasker track; we expressed the count as ratio over the number of regions for each dataset. We also extracted the ratio of methylome that overlapped each class of repeats and performed a Chi-square test. We could not detect any significant enrichment.

#### Off-Target Predictions

Putative off-target sites of the *B2M*-TALEs or of the *B2M*-CRISPR were predicted using Target Finder from TALE-NT suite (Doyle et al., 2012) or CRISPR design suite (Hsu et al., 2013), respectively. For every putative off-target region, we looked at the closest TSS as well as the closest methylated region. We considered a true off-target effect a region associated either to a gene regulated with FDR < 0.01 and a distance to TSS smaller than 10Kb or a methylated region regulated with FDR < 0.01 and a distance lower than 1 Kb.

#### Western Blot Analyses

K-562 cells were transfected with plasmids encoding for either TALE- or dCas9-based ETRs (V5- or HA-tagged, respectively). Cells were harvested and lysed at the indicated time points in RIPA Buffer supplemented with Protease Inhibitor Cocktail (Roche). Samples were sonicated using a Diagenode BioruptorPico and protein concentration was measured using Biorad Protein Assay Reagent (Biorad) and an Eppendorf BioPhotometer. The cell lysate was fractionated using 8% SDS polyacrylamide gel. Proteins were transferred to a PVDF membrane using an iBlot System (Invitrogen). TBS-T (0.1% tween) with 5% BSA was used for all the blocking and antibody dilutions. Membranes were incubated with the following primary antibodies: mouse monoclonal anti-V5 tag antibody (1:1000, Abcam ab53418) or mouse monoclonal anti-HA tag antibody (1:1000, Sigma-Aldrich, cat.no.11583816001) for 16h at 4°C; rabbit polyclonal anti-CALNEXIN (1:3000, Genetex, GTX13504) for 1h at RT. After washing, membranes were incubated with an anti-mouse or an anti-rabbit IgG, HRP-conjugated antibody (1:10000, GE Healthcare NAP9310V or 1:20000, GE Healthcare NA93AV, respectively) for 1h at RT, then washed with TBS-T for 15 min and finally visualized by Luminata Forte Western HRP substrate (Amersham). Images were captured using a ChemiDoc Touch Imaging System (Bio-Rad) and processed using ImageLab software (Bio-Rad).

### Quantification and Statistical Analysis

Flow cytometry raw data were analyzed using FlowJo (FLowJo LLC) or FSC Express 4 (De Novo software). Raw data from real-time gene expressions and ChIP-qPCRs were analyzed using GraphPad Prism 7 (GraphPad Software). When indicated in the figure legends, statistical significance was evaluated using GraphPad Prism 7 (GraphPad Software) and unpaired t test, with the exception of Figure 4G (1-tail paired t test) and Figure S7C (chi-square test). Statistical significance of the data is indicated as follows: ^∗^p < 0.05; ^∗∗^p < 0.01; ^∗∗∗^p < 0.001; ^∗∗∗∗^p < 0.0001. Data presented in Figure S5G were clustered using DBSCAN algorithm implemented in python sklearn library with the following parameters: eps = 0.25, min_samples = 3).

### Data and Software Availability

#### Data Resources

The accession number for the MeDIP-seq and RNA-seq experiments reported in this paper GEO: GSE81826.

## Author Contributions

A.A. designed and performed research, analyzed data, and wrote the paper; A.M. designed and performed research, analyzed data, and wrote the paper; P.C. assisted with research; M.B. assisted with research; D.C. analyzed the MeDIP and RNA-seq experiments; L.N. designed, supervised, and coordinated research and wrote the paper with input from all authors; A.L. conceived the study, designed, supervised, and coordinated research and wrote the paper with input from all authors. L.N. and A.L. share senior authorship.

## Supplemental Information

Table S1. Amino Acid Sequences of the Effector Domains, Related to Figures 1 and 3 Provided as a media file.

Table S2. List of the TaqMan Gene Expression Assays Used in this Study, Related to Figure 1 Provided as a media file.

Table S3. Nucleotide Sequences of the CRISPRs and the TALEs Target Sites, Related to Figure 4 Provided as a media file.

Table S4. List of Primers Used in this Study, Related to the Figure 6 Provided as a media file.

Table S5. Summary of the RNA-Seq Analysis, Related to the Figure 7 Summary results of the transcriptome profiling of TALE-silenced versus mock-treated cells and dCas9-silenced versus mock-treated cells by RNA-seq. For each gene, we report the log fold change, nominal p value, and false discovery rate (FDR) resulting from edgeR analysis (for more details on these analyses refer to STAR Methods). For each condition, an additional column flags genes that are significant under an FDR lower than 0.01. B2M-IRES-tdTomato gene is highlighted in light blue. Provided as a media file.

Table S6. Summary of the MeDIP-Seq Analysis, Related to the Figure 7 Summary results of the methylome profiling of TALE-silenced versus mock-treated cells and dCas9-silenced versus mock-treated cells by MeDIP-seq. For each analyzed region, we report the log fold change, nominal p value, and false discovery rate (FDR) resulting from edgeR analysis after CQN normalization (for more details on these analyses refer to STAR Methods). For each condition, an additional column flags genes that are significant under an FDR lower than 0.01. Target region for silencing is highlighted in light blue. Provided as a media file.

Table S7. Off-Target Analysis, Related to the Figure 7 Putative off-target sites of the ETRs were predicted as described in STAR Methods. For each putative off-target, we report the closest methylated region and the closest gene. Fold changes and statistical analyses refer to TALE-silenced versus mock-treated cells or dCas9-silenced versus mock-treated cells (Tables S5 and S6). Provided as a media file.
